# Molecular dynamics simulations of the secondary-binding site in disaccharide-modified glycopeptide antibiotics

**DOI:** 10.1038/s41598-022-10735-6

**Published:** 2022-04-30

**Authors:** Olatunde P. Olademehin, Kevin L. Shuford, Sung J. Kim

**Affiliations:** 1 Department of Chemistry and Biochemistry, Baylor University, Waco, TX 76706 USA; 2 Department of Chemistry, Howard University, Washington, DC 20059 USA

**Keywords:** Biophysical chemistry, Mathematics and computing

## Abstract

Oritavancin is a semisynthetic glycopeptide antibiotic used to treat severe infections by multidrug-resistant Gram-positive pathogens. Oritavancin is known to be a thousand times more potent than vancomycin against Gram-positive bacteria due to the additional interactions with bacterial peptidoglycan (PG) facilitated by a secondary-binding site. The presence of this secondary-binding site is evident in desleucyl-oritavancin, an Edman degradation product of oritavancin, still retaining its potency against Gram-positive bacteria, whereas desleucyl-vancomycin is devoid of any antimicrobial activities. Herein, using explicit solvent molecular dynamics (MD) simulations, steered MD simulations, and umbrella sampling, we show evidence of a secondary-binding site mediated by the disaccharide-modified hydrophobic sidechain of oritavancin interactions with the pentaglycyl-bridge segment of the PG. The interactions were characterized through comparison to the interaction of PG with chloroeremomycin, vancomycin, and the desleucyl analogs of the glycopeptides. Our results show that the enhanced binding of oritavancin to PG over the binding of the other complexes studied is due to an increase in the hydrophobic effect, electrostatic and van der Waals interactions, and not the average number of hydrogen bonds. Our ranking of the binding interactions of the biomolecular complexes directly correlates with the order based on their experimental minimum inhibitory concentrations. The results of our simulations provide insight into the modification of glycopeptides to increase their antimicrobial activities or the design of novel antibiotics against pathogenic Gram-positive bacteria.

## Introduction

**Figure 1:**
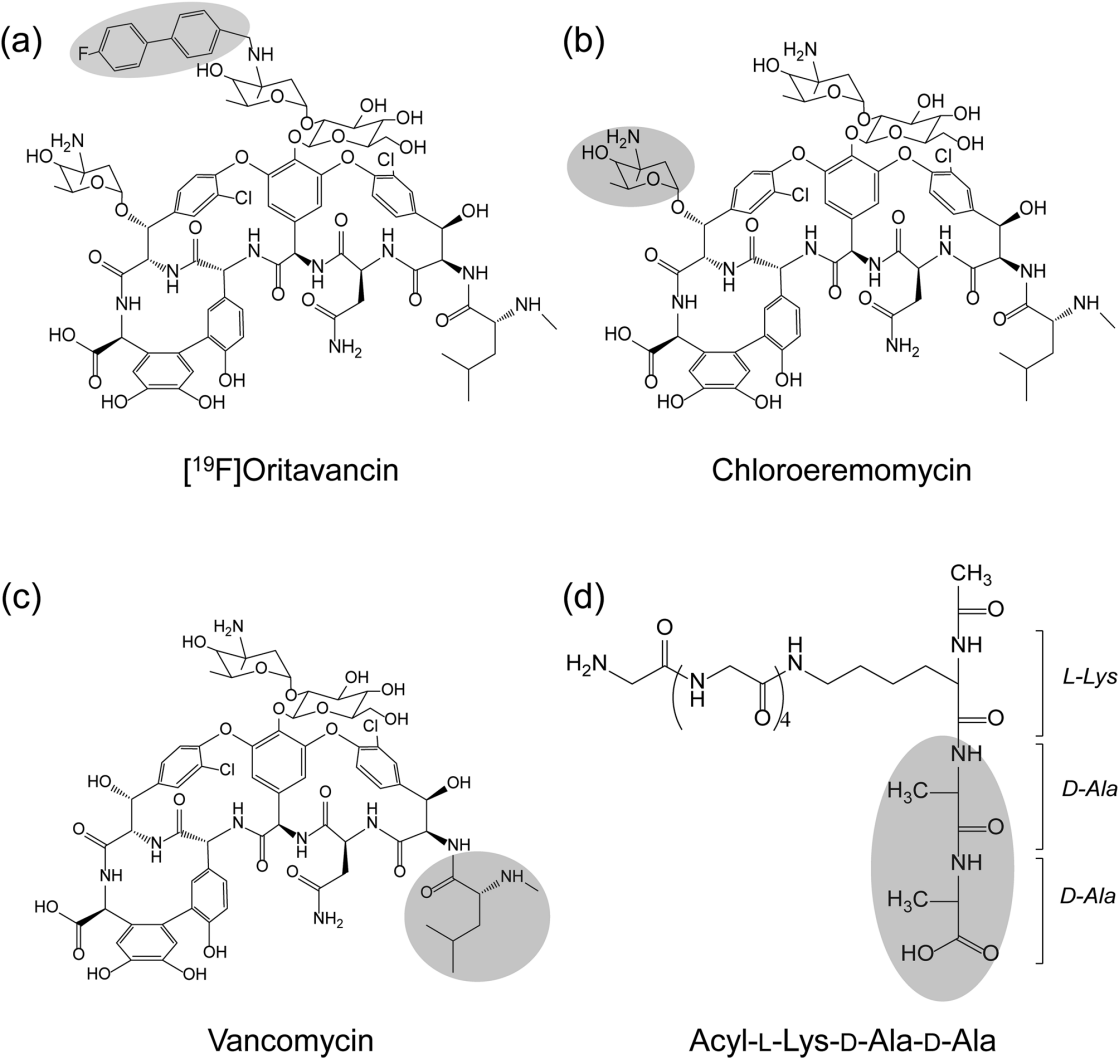
The chemical structures of the glycopeptide antibiotics and a PG-repeat unit. (**a**) Chemical structure of [^19^F]oritavancin with the biphenyl moiety highlighted in grey. (**b**) Chemical structure of chloroeremomycin with the 4-epi-vancosamine highlighted in grey. (**c**) Chemical structure of vancomycin with the first amino acid residue, *N*-methylleucine (grey circle). The cleavage of the *N*-methylleucine from [^19^F]oritavancin, chloroeremomycin, and vancomycin by Edman degradation results in desleucyl-[^19^F]oritavancin, desleucyl-chloroeremomycin, and desleucyl-vancomycin, respectively. (**d**) Chemical structure of the peptide portion of the PG-repeat unit in *S. aureus* with amino acid sequence acyl-l-Lys(Gly_5_)-d-Ala-d-Ala. A pentaglycine is attached to the ɛ-nitrogen of the l-Lys. The primary-binding site of the glycopeptide antibiotics binds to the d-Ala-d-Ala of the PG-repeat unit (grey oval). The PG disaccharide *N*-acetylglucosamine-*N*-acetylmuramic acid does not participate in the binding and thus was removed from the glycopeptide–PG complex.

Glycopeptides consist of powerful antibiotics that are in clinical use including vancomycin, teicoplanin, telavancin, dalbavancin, and oritavancin (Fig. 1). All glycopeptide antibiotics target bacterial cell wall biosynthesis by binding to the d-Ala-d-Ala terminus^1^ of the membrane-bound peptidoglycan (PG) precursor, lipid II. Lipid II refers to a PG-repeat unit attached to the lipid transporter (C_55_). The PG-repeat unit in *Staphylococcus aureus* consists of a disaccharide (GlcNAc-MurNAc), a pentapeptide-stem structure of l-Ala-d-iso-Glu-l-Lys-d-Ala-d-Ala, and a pentaglycine attached to the ɛ-nitrogen side of the Lys (Fig. 1d). Glycopeptide antibiotic binding to lipid II prevents the transglycosylation step of PG biosynthesis, which is required to regenerate the lipid transporter. Hence, the addition of vancomycin to *S. aureus* during the growth results in the accumulation of Park’s Nucleotide which is a cytoplasmic PG-precursor^2^. Since C_55_ is found in a surprisingly low number of copies per bacterium^3–5^ and it is also a shared transporter used in wall-teichoic acid biosyntheses, vancomycin binding to lipid II is an effective means of inhibiting both PG and wall-teichoic acid biosyntheses in *S. aureus*^6,7^.

In vancomycin-resistant pathogens, the d-Ala-d-Ala terminus of the PG stem is either cleaved or modified to prevent the glycopeptide binding. The d-Ala-d-Ala terminus modification to d-Ala-d-Ser is found in low-level vancomycin-resistant bacteria^8,9^, and modification to d-Ala-d-Lac is found in high-level vancomycin-resistant enterococci (VRE)^8,10–12^. The depsipeptide substitution reduces the vancomycin binding affinity (K_d_) by a thousand fold from 1 μM for a tripeptide acyl-l-Lys-d-Ala-d-Ala to 1 mM for a tripeptide acyl-l-Lys-d-Ala-d-Lac, and increases the minimal inhibitory concentration (MIC) from 1 µg/ml against vancomycin-susceptible bacteria to greater than 1000 µg/ml against VRE. The replacement of the d-Ala-d-Ala by d-Ala-d-Lac substitutes an amide with an ester bond which replaces one of the hydrogen bonds with an electrostatic repulsion between the lone pair of a carbonyl oxygen atom at the 4th position of vancomycin aglycon and the ester oxygen atom on the d-Ala-d-Lac^13,14^*.*

A series of chemical modifications by alkylation of the amine sugar (l-4-epi-vancosamine) of chloroeremomycin (Fig. 1b) with a hydrophobic group has led to the discovery of second-generation lipoglycopeptide antibiotics including oritavancin which exhibits potent activity against the VRE^15,16^. Chloroeremomycin is the parent compound of oritavancin which differs from vancomycin by an additional 4-epi-vancosamine attached to the 6th amino acid of the heptapeptide core. While chloroeremomycin is only four times more potent than vancomycin, oritavancin shows a thousand-fold increase in the efficacy against the VRE. Oritavancin’s potent antimicrobial activity against the VRE came as a surprise because the 4-chloro-biphenyl is attached to the vancosamine which is positioned far from the d-Ala-d-Ala binding site. Thus, an alkylated hydrophobic sidechain of oritavancin, despite a thousand-fold increase in activity, does not improve the glycopeptide binding affinity to the peptides terminating in either d-Ala-d-Ala or d-Ala-d-Lac. The dissociation constants for vancomycin and oritavancin to acyl-d-Ala-d-Lac are both on the order of 1 mM, suggesting that oritavancin has a distinct mode of action that involves more than d-Ala-d-Ala binding. Furthermore, oritavancin exhibits the following unique antimicrobial properties that are consistent with a new mode of action: (1) while vancomycin is a biostatic agent, oritavancin is bactericidal achieving 3-log kill (99.9% reduction) within an hour of drug addition^17,18^, (2) unlike vancomycin, oritavancin retains the potent killing kinetic against the VRE and vancomycin-resistant *S. aureus*^18^, and (3) while the Edman degradation product of vancomycin is devoid of activity, the Edman degradation product of oritavancin retains its potent activity^19–21^*.*

The alkylated hydrophobic adducts of oritavancin and oritavancin-like lipoglycopeptide have been thought to act as membrane anchors through insertion into the bacterial lipid bilayer to localize the drug at the site of PG biosynthesis and to promote drug dimerization^22^. This is corroborated by a strong correlation between the lipophilicity indices of the alkylated hydrophobic adducts of the lipoglycopeptide and the MIC of the drug^23^. The co-localization of glycopeptide to its substrate at the membrane by membrane anchor is energetically favorable for intermolecular interaction and thereby overcoming weak binding to depsipeptide terminating lipid II^24^. This is supported by an observation that chloroeremomycin dissociation constant to a depsipeptide d-Ala-d-Lac in solution (1 mM)^25^ decreases to 4 µM when a PG-mimic, docosanoyl-Gly-Ala-Glu-Lys-d-Ala-d-Lac, is anchored to the phosphatidyl-choline vesicle^26^. Drug dimerization is also thought to enhance the depsipeptide substituted lipid II bindings through positive cooperativity^24^. In solution, oritavancin dimerizes strongly with a dimerization constant approximately a thousand times greater than vancomycin^23,27^. Hence, combined drug dimerization and membrane anchoring are thought to be the mode of action that pervades in all lipoglycopeptides to overcome the vancomycin resistance in VRE.

Although in vitro assays have shown that lipoglycopeptides dimerizes in solution and binds to lipid vesicles, in situ lipoglycopeptides exhibit a different mode of action^20,28–34^. Solid-state nuclear magnetic resonance (NMR) characterization of the ^19^F-labeled oritavancin (Fig. 1a) bound to intact whole cells of *S. aureus* confirmed that [^19^F]oritavancin preferentially binds to the PG as a monomer, not as a dimer, and oritavancin does not localize to the lipid bilayer of the bacterial membrane^29^. Even when [^19^F]oritavancin was added to the protoplasts of *S. aureus*, where the cell wall is enzymatically removed using lysostaphin to leave only a thin layer of nascent PG, oritavancin is still found bound to the PG but not to the membrane^33^. The absence [^19^F]oritavancin membrane anchoring in situ was determined by ^31^P{^19^F} rotational-echo double resonance (REDOR) NMR, positioning the ^19^F of oritavancin at least 14 Å or further away from the phosphate found in the lipid headgroup^26^. Solid-state NMR characterization of oritavancin and other lipoglycopeptide antibiotics with a varying hydrophobic sidechain length bound to intact whole cells has revealed that the positions of the drug hydrophobic sidechain are closely associated with the crosslinked PG-bridge structure^28,32,34^. The hydrophobic sidechain of lipoglycopeptide is thought to form a secondary-binding^34^ to target partially crosslinked PG template to prevent the maturation of the nascent PG by inhibiting transpeptidase activity^30^. Hence, unlike vancomycin which inhibits transglycosylase activity, secondary-binding site in oritavancin enabled dual inhibition of both transglycosylase and transpeptidase activities in *S. aureus*^7,20,30,35,36^*.*

The significance of the secondary-binding site is evident when des-*N*-methylleucyl-oritavancin (desleucyl-oritavancin), an Edman degradation product of oritavancin, was complexed to whole cells of *S. aureus*^30^. For vancomycin, Edman degradation results in des-*N*-methylleucyl-vancomycin with the damaged d-Ala-d-Ala binding cleft which does not bind to PG and is devoid of any antimicrobial activities. However, desleucyl-oritavancin^30^ and Edman degradation products of oritavancin-like vancomycin^21^ retain potent antimicrobial activities. Despite the damage to the primary-binding site, the secondary-binding site in desleucyl-oritavancin^30^ and desleucyl-chloro-biphenyl vancomycin^31^ have been shown to enable binding to PG in intact whole cells of *S. aureus*. Furthermore, the addition of desleucyl-oritavancin to *S. aureus* during growth has been shown to inhibit transpeptidase activity of cell wall biosynthesis which was confirmed by solid-state NMR^20^ and by liquid chromatography-mass spectrometry analysis of the muropeptides^35^. The inhibition of transpeptidase activities by desleucyl-oritavancin showed that the secondary-binding site targeted the peptidoglycan template to induce cell wall disorder and to interfere with cell wall maturation. Despite the significance and relevance of the secondary-binding site in oritavancin, the structure and conformation of the oritavancin–PG complex remain unknown. This is because the secondary-binding site targets the crosslinked PG in the cell wall, which is not amenable to either x-ray diffraction or solution-state NMR for structural determination.

In this study, we investigate the role of the secondary-binding site in oritavancin complexed to the PG-repeat unit using molecular dynamics (MD) simulations, center-of-mass (COM) pulling simulations, and umbrella sampling. The simulations were carried out for the binding of PG with oritavancin, chloroeremomycin, vancomycin, and their desleucyl analogs (Fig. 1). Our results show evidence of a secondary-binding site mediated by the disaccharide-modified hydrophobic sidechain of oritavancin interactions with the pentaglycyl bridge segment of the PG. These interactions contributed to the significant enhancement in oritavancin binding to PG compared to other glycopeptide antibiotics that do not have a secondary-binding site (chloroeremomycin and vancomycin). For the MD simulations, the experimentally determined solid-state NMR distance constraints^20,30,37^ were utilized for the glycopeptide–PG and desleucyl-glycopeptide–PG complexes^20,30,37^*.*

## Methods

### Molecular dynamics (MD) simulations

The initial structure of [^19^F]oritavancin complexed with the PG-repeat unit (Ori–PG) was based on a computational model reported in a previous study^37^. The Ori–PG complex was modified based on the solid-state NMR structure^29,38^ with GaussView 6.11 and optimized with a Gaussian 16 program package using density functional (DFT) calculations at the B3LYP/6-311G (d,p) level of theory. The energy minimized structure obtained was modified to give the other complexes used in the simulation. The chloroeremomycin–PG (CEremo–PG) complex, which represents chloroeremomycin complexed to PG-repeat unit, is the Ori–PG complex modified by removing the biphenyl moiety attached to the vancosamine sugar at the 4th residue of the heptapeptide core. The vancomycin–PG (Vanco–PG), representing a complex of vancomycin and the PG-repeat unit, is obtained by modifying the Ori–PG complex through the removal of the biphenyl moiety on the vancosamine sugar at the 4th amino acid residue and the 4-epi-vancosamine attached to the 6th amino acid of the heptapeptide core. For each of the complexes, the desleucyl-glycopeptide–PG analogs are derived by removing the first amino acid residue of the aglycon, *N*-methylleucine. The initial topology files for the simulation system was generated through the online ParamChem/CGenFF-4.0 server (https://cgenff.paramchem.org/)^39^. The CHARMM36 force field was used for atom parameters.

Four independent simulations were carried out for each complex under periodic boundary conditions using GROMACS version 2018.3^40^. All the systems were solvated with transferable intermolecular potential with 3 points (TIP3P) explicit water molecules in a rhombic dodecahedral box for the equilibrium simulations, with the complex centered and placed at least 1.0 nm from the box edge. Sodium and Chlorine ions were added as charge-balancing ions to a concentration of 0.15M to mimic physiological conditions. The simulation boxes were subjected to a maximum of 50,000 steps of the steepest descent energy minimization to guarantee the maximum force acting on each atom is below 1000 kJ/mol/nm. Thereafter, constant volume and temperature (NVT) equilibration was performed on the energy minimized system for 200 ps at a time-step of 1 fs using the leap-frog integrator and modified Berendsen thermostat for equilibrating the system to a temperature of 300 K. Initial velocities were assigned from a Maxwellian distribution. The NVT equilibration was followed by a constant pressure and temperature (NPT) equilibration for 10 ns with 2 fs time-step to stabilize the system’s pressure at 1 bar using Parrinello-Rahman barostats with the compressibility of 4.5 × 10^–5^ bar^−1^. The isotropic position scaling protocol was used to control the pressure. Long-range electrostatic interactions were modeled using the particle mesh Ewald (PME) model, and the length of all covalent bonds constrained with the linear constraint solver (LINCS) algorithm^41^. The molecular dynamics simulation was performed for 30 ns at a time-step of 2 fs and the output saved after every 10 ps for each simulation system. A 12 Å cut-off was used for short-range interactions. During the MD simulation, atomic pair distance constraints were applied based on solid-state NMR experiments^37^*.*

### Root-mean-square deviation (RMSD)

The rmsd was used to quantitatively measure the conformational difference between the structures of each complex along the simulation trajectories and a stable reference structure of the complex, after NVT and NPT equilibration, to estimate the structural similarities. This was achieved by least-squares fitting of the dynamic structure to the reference structure using GROMACS built-in functions. The rmsd equation for a molecular structure represented by a cartesian coordinate vector ${\mathbf{r}}_{i} (i=1-N)$ of N atoms is illustrated by

1 $$\mathrm{RMSD}= \left[\frac{1}{\mathrm{M}} \sum_{i=1}^{N}{m}_{i}({\mathbf{r}}_{i}^{t}-{\mathbf{r}}_{i}^{0}{)}^{2}\right]^{1/2},$$

where $\mathrm{M}= \sum_{i=1}^{N}{m}_{i}$, ${\mathbf{r}}_{i}^{t}$ is the position of atom $i$ along the trajectory at time $t$, ${\mathbf{r}}_{i}^{0}$ is the position of atom $i$ for the stable reference structure, and ${m}_{i}$ is the mass of atom $i$. The rmsd values for each of the glycopeptide–PG complex were obtained by comparing their configuration at $t=0$ with the complex dynamic structure along the trajectories during MD simulation.

### Hydrogen bond analysis

The stability of the moderate hydrogen bonds formed between the glycopeptide and PG-repeat unit in each complex along the trajectory of the MD simulation was monitored using the hydrogen bond analysis. The hydrogen bond profiles for the various complexes were obtained using the g_hbond utility in GROMACS, considering all possible hydrogen bond donors and acceptors. The criteria for the hydrogen bond formation are based on the donor–acceptor distance of 3.0 Å and the angle cut-off of 20° within a linear configuration. The average number of hydrogen bonds formed in a particular complex during the simulation can be comparable to the stability of the complex along the simulation trajectories.

### Binding free energy calculations

The binding free energy of the different glycopeptide–PG complexes considered in the MD simulation study was obtained using both an end-point and pathway methods of binding energy computation. Specifically, we utilized the molecular mechanics Poisson–Boltzmann surface area (MM/PBSA) for the end-point binding energy determination and the umbrella sampling method for the pathway binding energy computation.

### Molecular mechanics Poisson–Boltzmann surface area (MM/PBSA) binding energy determination

In the MM/PBSA method, the free energy of binding, ${\Delta G}_{bind}$, of the PG to the glycopeptide (gly) to form a complex is given by the equation

2 $${\Delta G}_{bind}={G}_{complex}- {G}_{gly}-{G}_{PG}.$$

The ${\Delta G}_{bind}$ can be decomposed into the contributions from the different interactions within the system as shown below

3 $${\Delta G}_{bind}= \Delta {E}_{MM}+\Delta {G}_{sol}-T\Delta S$$

in which

4 $$\Delta {E}_{MM}=\Delta {E}_{int}+\Delta {E}_{elec}+\Delta {E}_{vdW},$$

5 $$\Delta {G}_{sol}= \Delta {G}_{polar}+\Delta {G}_{nonpolar},$$

where $\Delta {E}_{MM}$ is the change for the gas phase molecular mechanics (MM) energy, comprised of the changes in internal energies, $\Delta {E}_{int}$, (bond, angle, and dihedral energies), electrostatic energies $\Delta {E}_{elec}$, and the van der Waals energies $\Delta {E}_{vdW}$; $\Delta {G}_{sol}$ is the sum of the polar (electrostatic) solvation energy, $\Delta {G}_{PB}$, and the nonpolar contribution, $\Delta {G}_{nonpolar}$, between the solute and the continuum solvent. The polar contribution is calculated with the Poisson–Boltzmann model, while the nonpolar part is estimated with the solvent-accessible volume (SAV) model.

### Umbrella sampling binding energy determination

Umbrella sampling is a useful tool in analyzing macromolecular interactions, and the binding energy from umbrella sampling simulations is obtained from the potential of mean force (PMF)^42,43^. The umbrella sampling simulations of the glycopeptide–PG complex were performed following a center-of-mass (COM) pulling simulation to generate a series of initial configurations. Each of these configurations generated corresponds to a point in space where the PG is harmonically restrained at increasing COM distance from the glycopeptide through an umbrella biasing potential. The biasing force is illustrated with the equation

6 $$F= \frac{k}{2}\left[\zeta -\left({\zeta }_{0}+vt\right)\right],$$

where $v$ is the pulling velocity, $t$ is time, and k is the force constant. ${\zeta }_{0}$ is the reference coordinate point along the pulling path, and $\zeta$ is the coordinate point at a given time.

After the explicit solvent MD simulations, structures from the end of the trajectories were used for the pulling simulation. The glycopeptide complex was placed in a rectangular box with sufficient space for the PG to move along the z-axis during the pulling simulation and to satisfy the minimum image convention. Following solvation with TIP3P water and the addition of charge-balancing ions to a concentration of 0.15 M, the energy of the system was minimized with the steepest descent algorithm. Afterward, a 100 ps NPT equilibration at 2 fs time-step was performed using Berendsen weak coupling to maintain the pressure isotropically at 1.0 bar, with the complex heavy atoms position restrained. In the course of the pulling simulation, the restraints on the PG were removed while the glycopeptide remained as a stationary reference. The PG was pulled away from the glycopeptide along the z-axis for 500 ps at 2 fs time-step using a spring constant of 1000 kJ mol^−1^ nm^−2^ by applying a pulling rate of 0.01 nm ps^−1^. This pull rate was sufficient to maintain the integrity of the system. At the end of the pulling simulation, a COM distance of about 5.5 nm was achieved. Snapshots from the pulling simulation trajectories were taken as starting configurations for the umbrella sampling windows. A non-uniform window spacing ranging from 0.02 to 0.1 nm was used to ensure detailed sampling of configurations along the reaction coordinate and sufficient overlap of neighboring windows to obtain a continuous energy function. In total, about 50 windows were sampled on average. For the umbrella sampling, a 10 ns MD simulation was performed in each of the windows, resulting in a cumulative simulation time of 500 ns for one trial of each complex. The umbrella sampling simulations were carried out with the GROMACS version 2018.3. The weighted histogram analysis method (WHAM)^44^ method was used to extract the equilibrium data (PMF) from the nonequilibrium umbrella sampling windows. For each (desleucyl)glycopeptide–PG complex, the bootstrap method was used to estimate the error in the free energy of binding obtained from umbrella sampling.

## Results

### Hydrogen bonding, experimental MIC, and binding free energy

**Figure 2:**
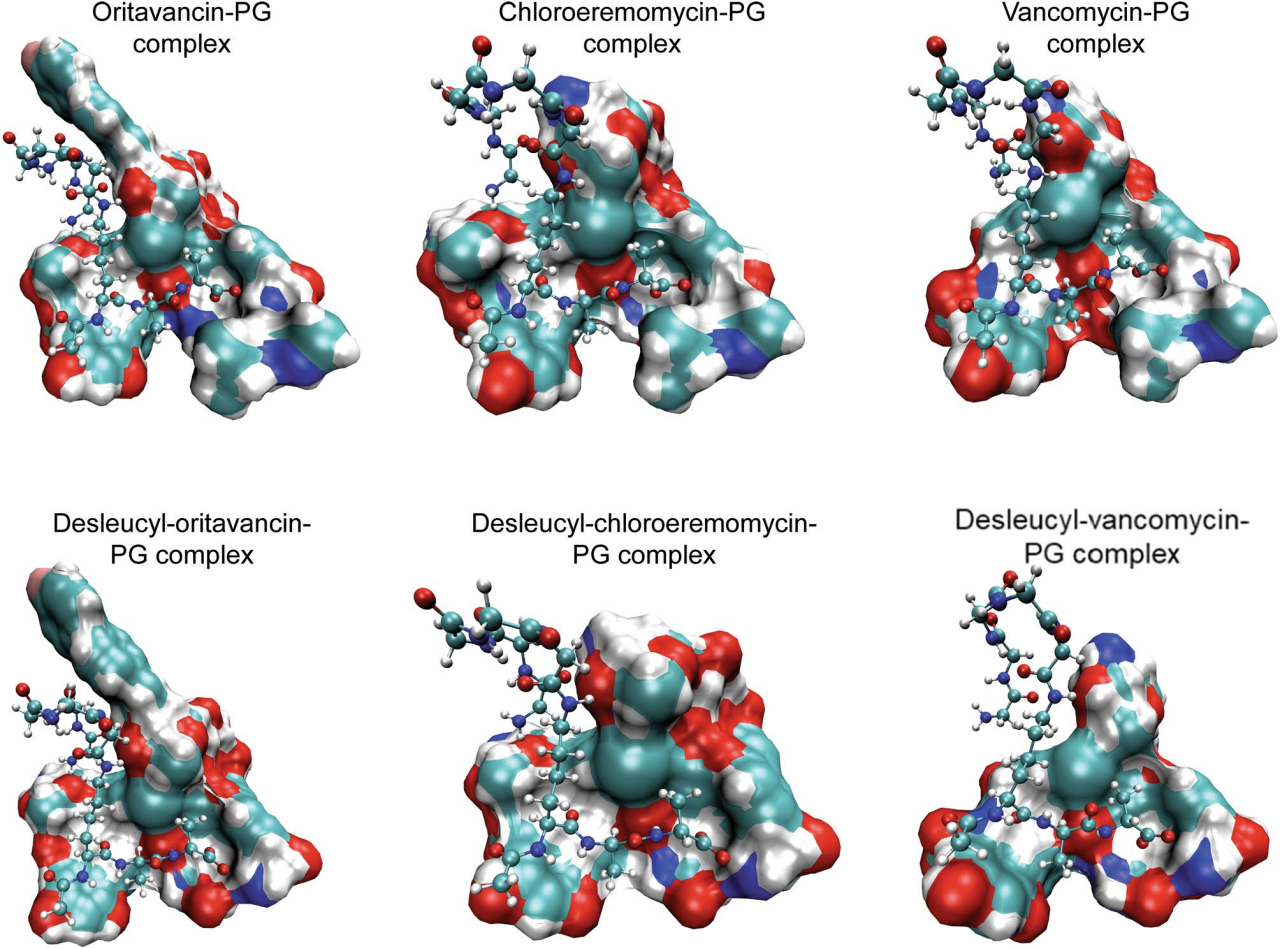
MD simulation model structures of the glycopeptide- and desleucyl-glycopeptide–PG complexes. The glycopeptides (top) and desleucyl-glycopeptides (bottom) are shown as a visual molecular dynamics (VMD)^45^ solid surf model using a probe radius of 1.4 Å, while the CPK model represents the PG (Fig. 1d). The glycopeptide–PG complexes (top) include [^19^F]oritavancin–PG complex (top left), chloroeremomycin–PG complex (top middle) and vancomycin–PG (top right). The desleucyl-glycopeptide–PG complexes (bottom) include desleucyl[^19^F]oritavancin–PG complex (bottom left), desleucyl-chloroeremomycin–PG complex (bottom middle), and desleucyl-vancomycin–PG (bottom right).

For each glycopeptide–PG complex (Fig. 2), the calculated average number of hydrogen bonds, free energy of binding, and the measured minimum inhibitory concentrations (MIC) of glycopeptides against *S. aureus*^30^ are shown in Table 1. The heptapeptide-core structure of glycopeptide constitutes a primary-binding site that binds to the d-Ala-d-Ala dipeptide segment of the PG. The strength of this interaction is governed by electrostatic hydrogen bonding such that when vancomycin binds to the PG of vancomycin-susceptible *S. aureus*, five stable hydrogen bonds are formed between the heptapeptide-core structure of vancomycin and the d-Ala-d-Ala of the PG. Any chemical modification, to either glycopeptide antibiotic or PG, that affects this interaction results in loss of antimicrobial activity. For example, in the case of vancomycin-resistant *S. aureus* where d-Ala-d-Ala of the PG is mutated to a d-Ala-d-Lac, the depsipeptide substitution removes one of the hydrogen bonds and replaces it with an electrostatic repulsion. This results in a 1000-fold reduction in the binding affinity of vancomycin to PG accompanied by a thousand-fold increase in the MIC. All glycopeptide–PG complexes studied, including Ori–PG, CEremo–PG, and Vanco–PG, show a minimum of five stable hydrogen bonds formed between the primary-binding pocket in glycopeptide to the PG (Table 1). In the case of desleucyl analogs of the glycopeptides that have a damaged primary-binding pocket due to the Edman degradation, the average number of stable hydrogen bonds between the damaged glycopeptide and PG is less than five.

**Table 1:** An average number of hydrogen bonds, experimental MIC^30^, and free energy of binding for the (desleucyl)glycopeptide–PG complexes.

Glycopeptide–PG complex	Average number of H-bonds	MIC^a^ (µM)	∆Gbind^b^ (Kcal/mol)	∆Gbind^c^ (Kcal/mol)
Ori–PG	5.50 ± 0.21	< 0.016	− 15.04 ± 0.66	− 125.89 ± 18.06
Des-Ori–PG	4.69 ± 0.16	0.30	− 14.95 ± 0.99	− 112.06 ± 20.98
CEremo–PG	5.91 ± 0.07	0.29	− 13.99 ± 0.64	− 104.36 ± 16.35
Des-CEremo–PG	4.89 ± 0.08	10.00	− 12.56 ± 0.73	− 91.96 ± 17.62
Vanco–PG	5.52 ± 0.37	0.84	− 11.07 ± 0.41	− 88.06 ± 18.14
Des-Vanco–PG	4.76 ± 0.07	> 673	− 8.45 ± 0.48	− 75.77 ± 17.77

^a^Experimental minimal inhibitory concentration.

^b^Binding energy from umbrella sampling method.

^c^Binding energy from MM/PBSA method.

**Figure 3:**
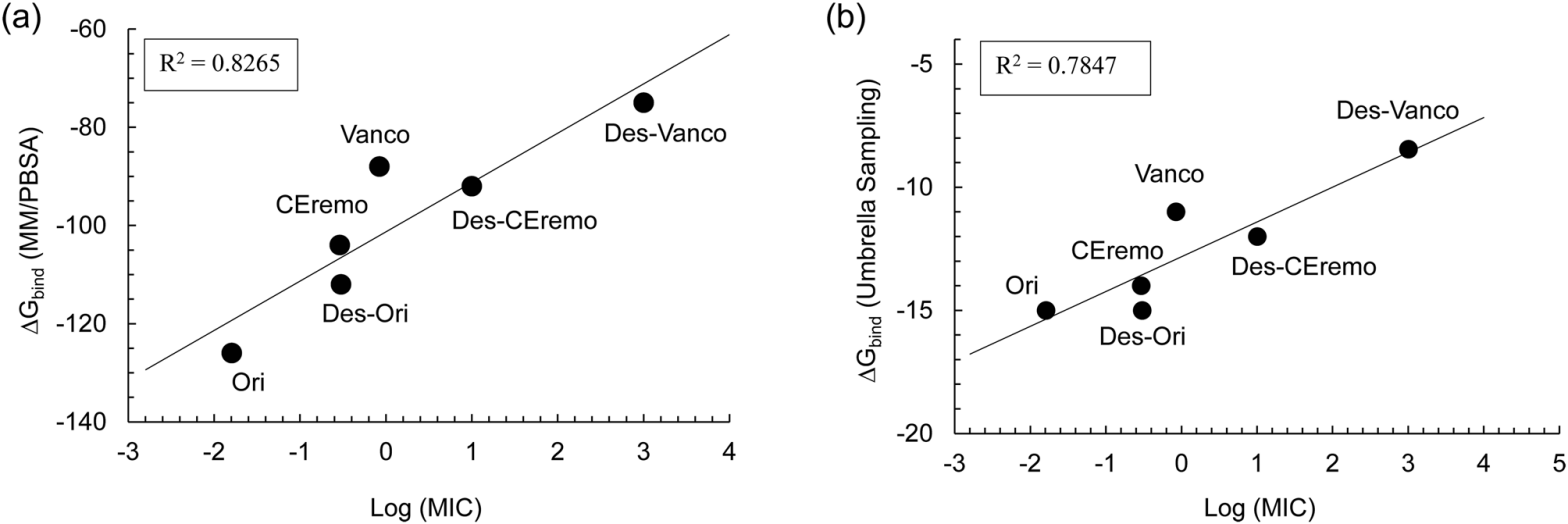
Plots of binding energy vs minimum inhibitory concentration for the (desleucyl)glycopeptide complexes. (**a**) Plots of MM/PBSA binding energy (*ΔG*_*bind*_) vs. Log (MIC). (**b**) Plots of umbrella sampling binding energy vs. Log (MIC).

The log of MIC values directly correlates with the free energy of binding for all the complexes (Fig. 3). The free energy of binding for the various complexes studied is calculated with both an end-point and a pathway method. The end-point free energy method is based on the molecular mechanics Poisson–Boltzmann surface area (MM/PBSA), while the more accurate pathway free energy calculation was obtained from the umbrella sampling (US) method. Both free energy calculation methods result in the same ranking of the stability of the (desleucyl)glycopeptide–PG complexes. Though the MM/PBSA free energy approach is considered less accurate than the umbrella sampling, it produced consistent results in ranking the binding interaction of biomolecular complexes, especially when similar ligands are involved in the binding^14,46^. From the binding free energy values, the stability of the (desleucyl)glycopeptide–PG complexes is ranked as follows: Ori–PG > Des-Ori–PG > CEremo–PG > Des-CEremo–PG > Vanco–PG > Des-Vanco–PG. The Des-Vanco–PG complex is the least stable complex with a ${\Delta G}_{bind}^{b}$ value of − 8.45 ± 1.80 kcal/mol while the Ori–PG complex is the most stable with a ${\Delta G}_{bind}^{b}$ of − 15.04 ± 1.74 kcal/mol, both in excellent agreement with the antimicrobial activities measured by the MIC^30^.

The decomposition of the MM/PBSA binding free energy into its electrostatic and van der Waals contribution to the total binding energy is given in Table 2. The increased binding of the Ori–PG complex compared to the other complexes can be seen as an overall increase in its electrostatic and van der Waals interactions. All the complexes that show comparatively increased binding based on the ΔG_bind_ values also show an increase in the electrostatic and van der Waals contribution to the free energy, except for the Des-CEremo–PG complex. The Des-CEremo–PG complex has a relatively lower van der Waals interaction than that of Vanco–PG but higher electrostatic interactions. From the error estimation shown for the van der Waals, electrostatic, and subsequently total binding energies of Des-CEremo–PG and Vanco–PG, there are no distinct differences in their energy values. Des-Vanco–PG with the least ΔG_bind_ value also has the least van der Waals and Electrostatic contribution to the total binding energy.

**Table 2:** MM/PBSA binding free energy decomposition.

Glycopeptide–PG complex	∆Evdw (Kcal/mol)	∆Eelec (Kcal/mol)	∆Gbind^a^ (Kcal/mol)
Ori–PG	− 62.08 ± 8.99	− 117.29 ± 9.86	− 125.89 ± 18.06
Des-Ori–PG	− 54.37 ± 9.04	− 111.90 ± 10.10	− 112.06 ± 20.98
CEremo–PG	− 47.76 ± 8.86	− 110.51 ± 10.17	− 104.36 ± 16.35
Des-CEremo–PG	− 40.09 ± 8.76	− 104.95 ± 10.43	− 91.96 ± 17.62
Vanco–PG	− 44.95 ± 8.46	− 101.69 ± 9.82	− 88.06 ± 18.14
Des-Vanco–PG	− 35.97 ± 8.37	− 99.35 ± 10.41	− 75.77 ± 17.77

^a^MM/PBSA approach.

### Root-mean-square deviation (RMSD) analysis

**Figure 4:**
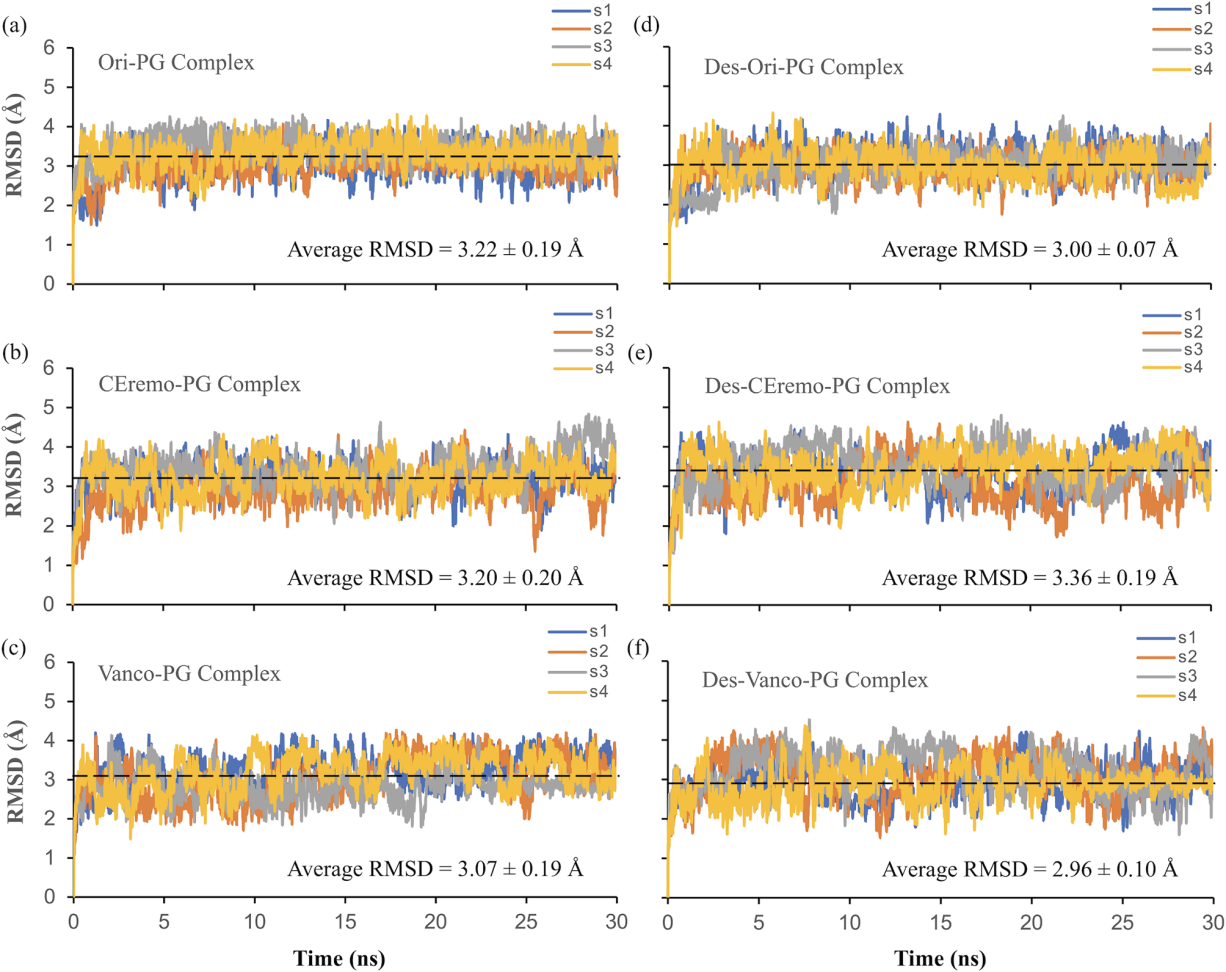
RMSD plots of MD simulations. The rmsd plots of the complexes of (**a**) [^19^F]oritavancin–PG, (**b**) chloroeremomycin–PG, (**c**) vancomycin–PG, (**d**) desleucyl[^19^F]oritavancin–PG, (**e**) desleucyl-chloroeremomycin–PG, and (**f**) desleucyl-vancomycin–PG. The rmsd plots are based on four independent simulations, labeled as s1, s2, s3, and s4. The average rmsd value for each complex is shown as a black dashed line.

The rmsd for each of the (desleucyl)glycopeptide–PG complex was calculated to gain insight into the stability of the complexes during the MD simulation (Fig. 4). The rmsd value is obtained by comparing the structures of the complex along the trajectories of the simulation with a stable reference structure. Generally, a relatively lower rmsd value and a narrow rmsd fluctuation pattern indicate a stable binding and lower flexibility between the (desleucyl)glycopeptide and PG. The rmsd values for all the glycopeptide–PG complexes (approximately 3.00 Å) are all within the error estimation for the four independent MD simulations performed. This closeness in rmsd values limits the use of the absolute rmsd values to rank the overall stability of all the complexes. From observing the various rmsd fluctuation pattern, Ori–PG and Des-Ori–PG complexes appear to be the most stable complexes due to their narrow and less flexible fluctuation patterns. The CEremo–PG and the Vanco–PG complexes also show comparatively lower fluctuation patterns when compared to that of Des-CEremo–PG and Des-Vanco–PG complexes, which both have more flexible fluctuation patterns.

The 2D rmsd (Å) contour plots were used to further characterize the binding distribution of the glycopeptide- and desleucyl-glycopeptide–PG complexes. The 2D contour plot compares the binding distribution between two complexes during MD simulation using their rmsd values. The presence of contour lines symmetric around a single attraction basin in a contour plot indicates the similarities of binding distribution between the two complexes that are being compared. Multiple attraction basins in a contour plot imply that the binding distribution of the two complexes compared is distinct.

**Figure 5:**
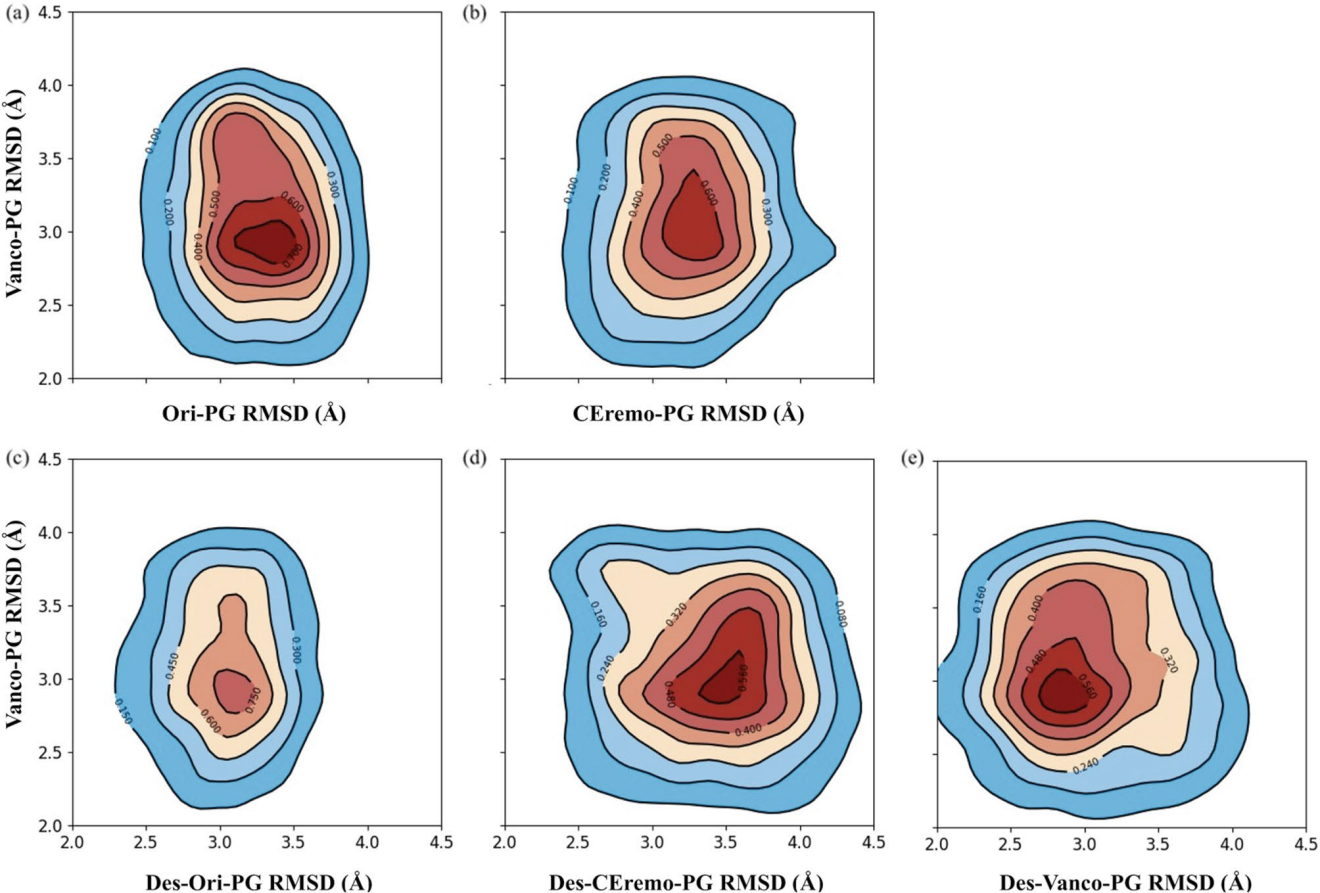
Comparative 2D rmsd (Å) contour plots of the Ori–PG complex relative to the other complexes. Comparative 2D rmsd contour plots of (**a**) Ori–PG vs. Ceremo–PG, (**b**) Ori–PG vs. Vanco–PG, (**c**) Ori–PG vs. Des-Ori–PG, (**d**) Ori–PG vs. Des-CEremo–PG, and (**e**) Ori–PG vs. Des-Vanco–PG complexes. The contour lines are the densities of trajectories located within an area. The region of the highest density of trajectories is denoted by the red color, while the blue colors represent regions of lower densities of trajectories.

The binding distribution of the Ori–PG complex was used as a reference to generate the 2D contour plots shown in Fig. 5. The binding distribution of the Des-Ori–PG complex (Fig. 5c), with its contour lines most symmetric around a single attraction basin at (3.1, 3.2) Å and centered on the highest density of joint distribution, indicates that the distribution is similar to that of Ori–PG complex despite the loss of its primary-binding site. We attribute this to the secondary-binding site which is present in both [^19^F]oritavancin and desleucyl[^19^F]oritavancin and contributes to the PG binding. This similarity in binding distribution between Ori–PG and Des-Ori–PG was also seen in their similar rmsd fluctuation pattern (Fig. 4a,d).

The next complex with a similar binding distribution to that of the Ori–PG complex is the CEremo–PG complex (Fig. 5a), based on the symmetrical contour lines. This is similar to the ranking of the complexes based on their binding energies and MIC values (Table 1) with Ori–PG > Des-Ori–PG > CEremo–PG. The 2D contour plots of Ori–PG vs. Vanco–PG, Des-CEremo–PG, and Des-Vanco–PG all show less symmetric contour lines, as well as a deviation from single attraction basins. All the complexes showed 2D contour plots with distinct binding distributions from that of Vanco–PG when the Vanco–PG was used as a reference for comparing other complexes (Supplementary Fig. S1).

### Force–time curve of center-of-mass pulling simulation

**Figure 6:**
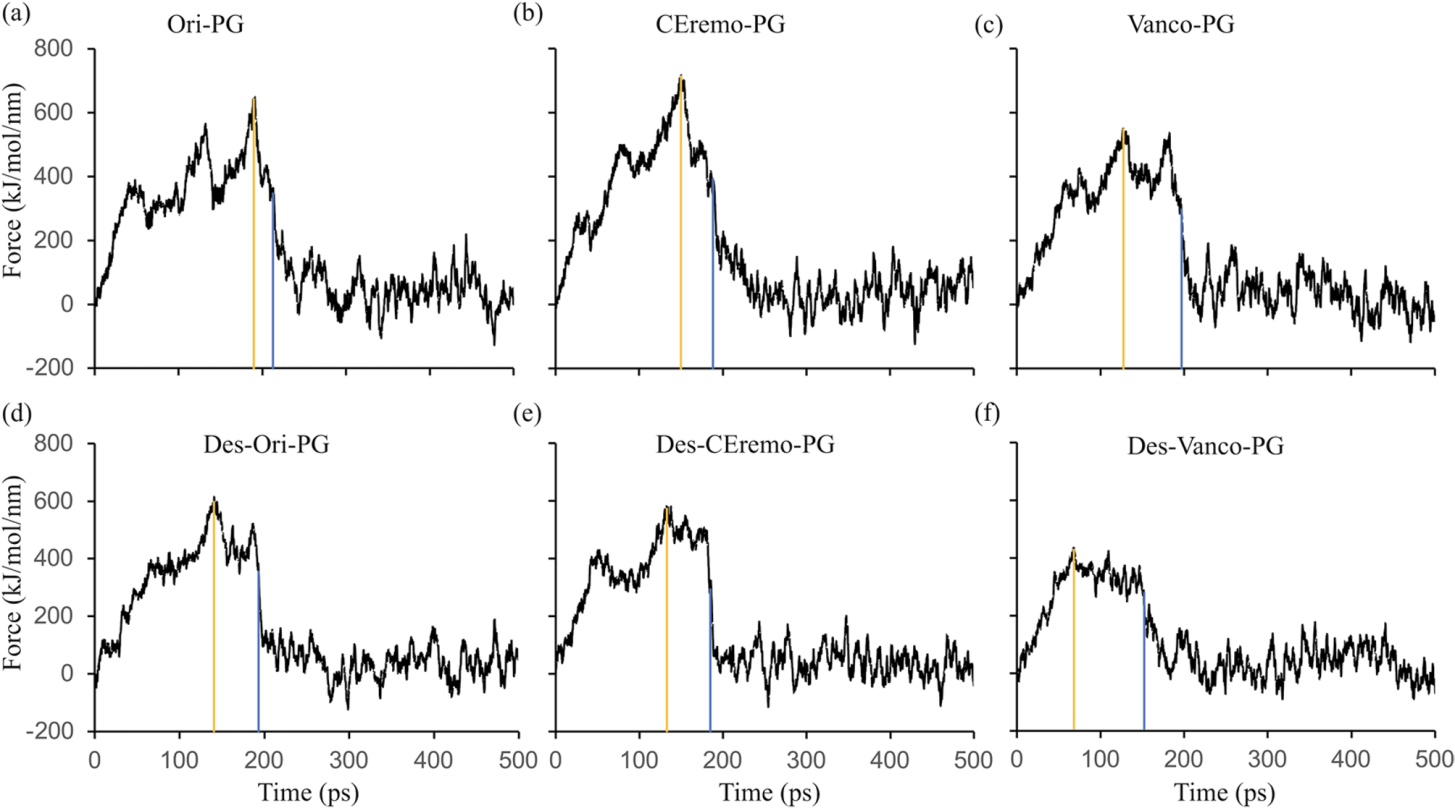
Plots of force vs time of the dissociation of the (desleucyl)glycopeptide and PG during center-of-mass (COM) pulling simulation. Plots of force vs time for (**a**) Ori–PG, (**b**) CEremo–PG, (**c**) Vanco–PG, (**d**) Des-Ori–PG, (**e**) Des-CEremo–PG, and (**f**) Des-Vanco–PG complexes. The yellow line in each figure corresponds to the point of maximum force and the blue line represents a major dissociation point between the (desleucyl)glycopeptide and PG.

The strength of the interaction between the (desleucyl)glycopeptide and PG can also be inferred from the force–time plot obtained from the COM pulling simulations for the various complexes (Fig. 6). The magnitude of the pull, maximum force (F_max_) required to pull out the bound PG from the glycopeptides, and the time required for dissociation of the complex can be used to infer the stability of the complex. This is especially true when the ligand-dissociation pathway is similar in the complexes compared^43^. In each glycopeptide–PG complex, the F_max_ required for pulling the PG away from the complex is higher than the corresponding desleucyl-glycopeptide–PG complex. Furthermore, a longer time is required to reach F_max_ and to achieve a major dissociation between the PG and the glycopeptide in glycopeptide–PG complex compared to the corresponding desleucyl-glycopeptide–PG complex. For example, the Ori–PG complex required a F_max_ of 645 kJ mol^−1^ nm^−1^ achieved in the duration of 91 ps (Fig. 6a, yellow line), and a total of 215 ps to reach the major point of dissociation between the PG and [^19^F]oritavancin (Fig. 6a, blue line). On the other hand, the Des-Ori–PG complex required a F_max_ of 616 kJ mol^−1^ nm^−1^ for the duration of 141 ps, and a major dissociation between the PG and desleucyl[^19^F]oritavancin was reached at 194 ps (Fig. 6d). The values for the F_max_, time to reach F_max_, and time to reach a major dissociation point for all the complexes studied are shown in Supplementary Table S1. The lower F_max_ and lesser time to reach a major dissociation point in the desleucyl-glycopeptide–PG complexes is due to the loss of binding in their primary-binding pockets. Of the (desleucyl)glycopeptide complexes, the Des-Vanco–PG required the minimum magnitude of F_max_ (437 kJ mol^−1^ nm^−1^) and took the least amount of time to reach the F_max_ (68 ps) and a major dissociation point (156 ps) (Fig. 6f). This is consistent with the Des-Vanco–PG complex having the least stability based on the binding energy and the minimum antimicrobial activity of Des-Vanco measured by the MIC (Table 1).

### Potential of mean force (PMF) from umbrella sampling simulation

**Figure 7:**
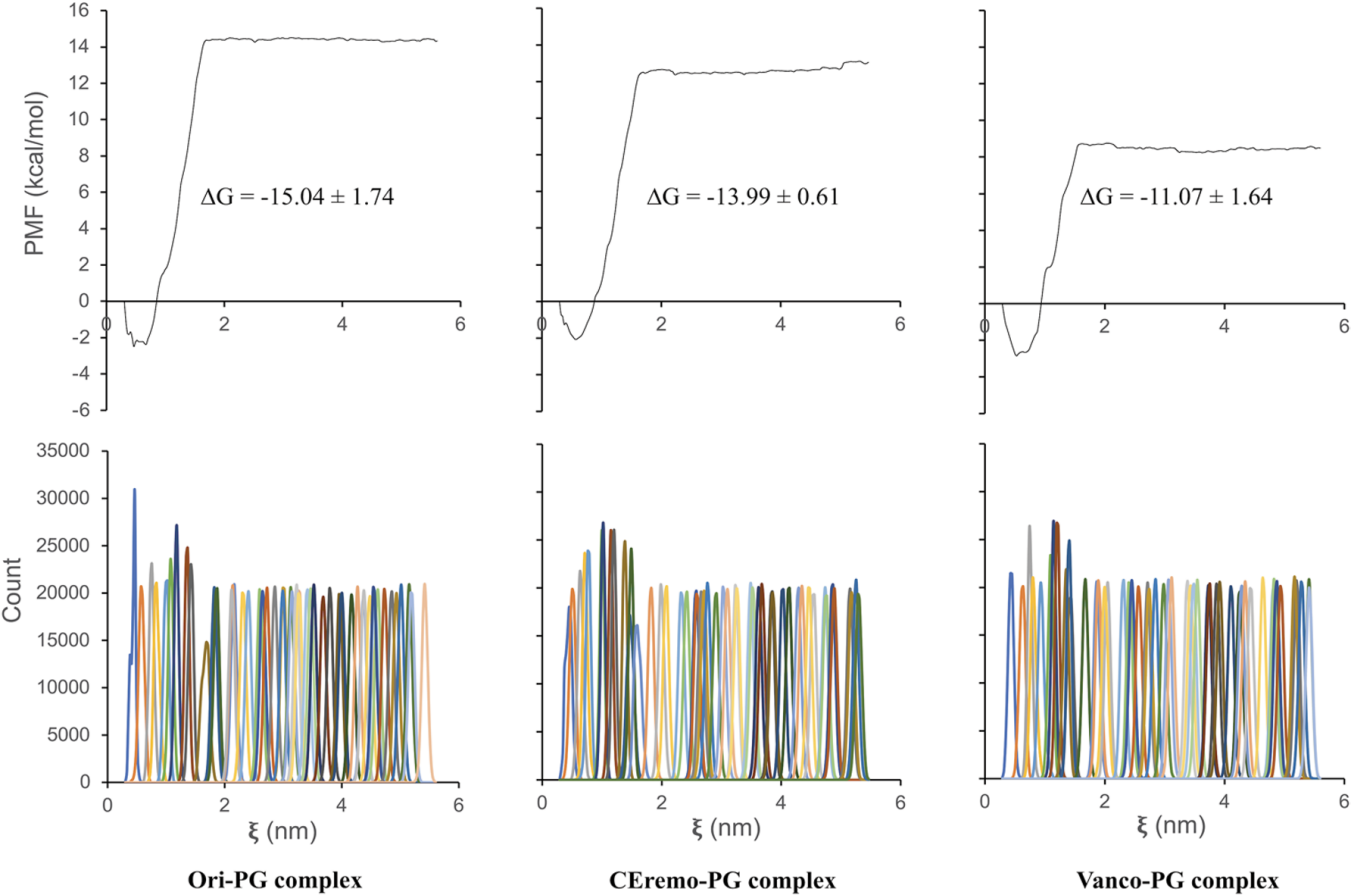
The potential of mean force (PMF) curve and corresponding umbrella histograms for complexes of [^19^F]oritavancin–PG (left), chloroeremomycin–PG (middle), and vancomycin–PG (right). The ΔG values of the complexes obtained from the PMF curve are shown as an inset. The PMF for the desleucyl-glycopeptide–PG complexes is included in the Supplementary Information.

The PMF along a reaction coordinate was obtained using umbrella sampling simulations^42^. Figure 7 shows the PMF leading to a ΔG_bind_, and the corresponding umbrella histograms for the glycopeptide–PG complexes from the weighted histogram analysis method (WHAM)^44^. Umbrella sampling is commonly performed following an explicit solvent MD simulation and a steered MD simulation, also known as COM pulling simulation, as an effective way to obtain the PMF along a reaction coordinate. About 50 sampling windows are used to run the umbrella sampling simulations, each corresponding to a 10 ns simulation.

**Figure 8:**
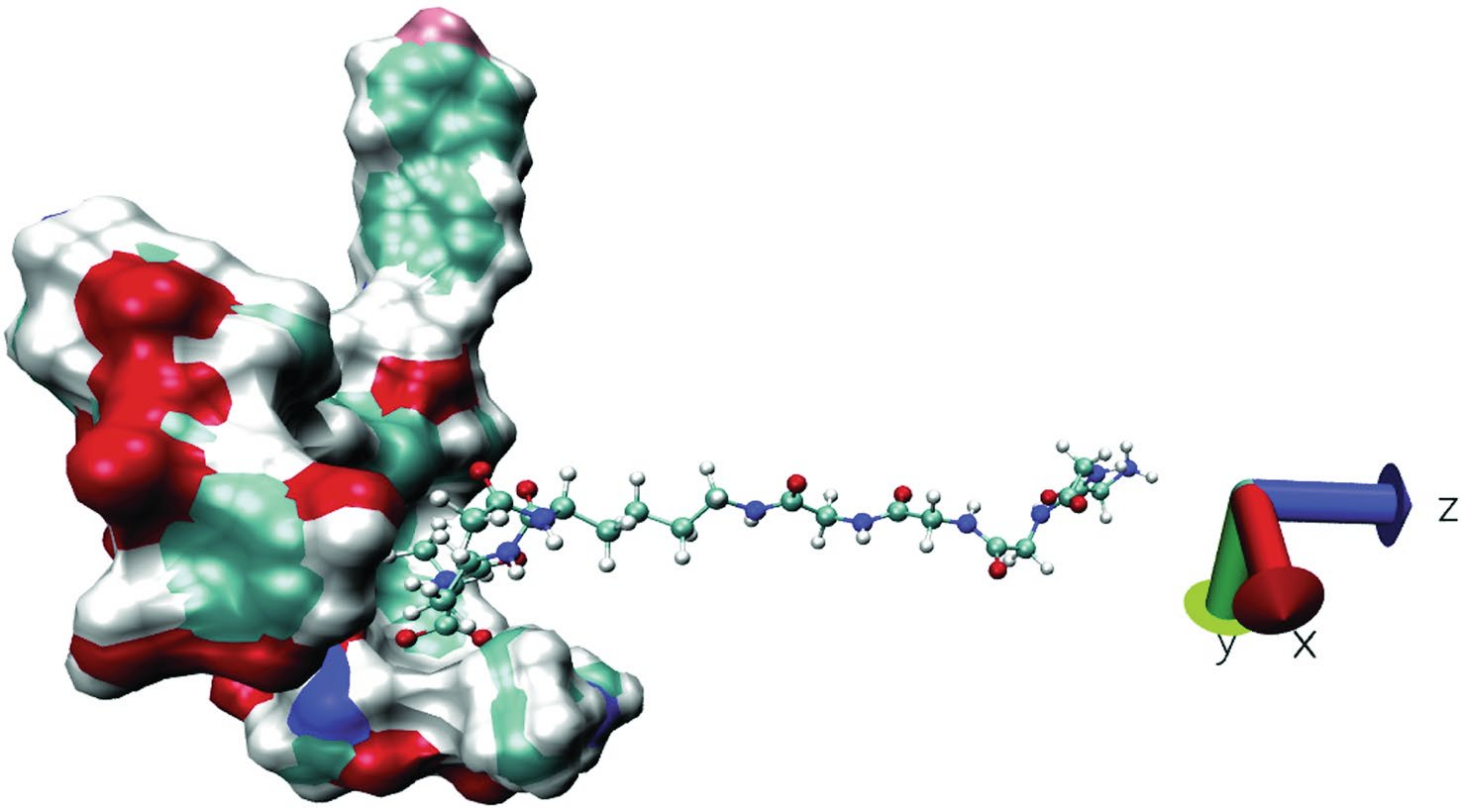
Representation of pulling simulation for (desleucyl)glycopeptide–PG complexes. The PG was pulled away from the (desleucyl)glycopeptide to generate series of configurations along the reaction coordinate (z-axis).

The COM pulling simulation was performed to a total COM distance of about 5.5 nm. The pulling bias was applied only in the z-dimension as shown in Fig. 8. The Ori–PG complex is the most stable complex from the PMF curves, among all the (desleucyl)glycopeptide–PG complexes studied, with a ΔG_bind_ value of − 15.04 ± 1.74 kcal/mol. The CEremo–PG complex has a ΔG_bind_ of − 13.99 ± 0.61 kcal/mol, and the ΔG_bind_ of the Vanco–PG complex is − 11.07 ± 1.64 kcal/mol (Fig. 7). The PMF curves for the desleucyl-glycopeptide–PG complexes are shown in Supplementary Fig. S2.

## Discussion

Solid-state NMR has been used to confirm the existence of a secondary-binding site in the binding of [^19^F]oritavancin to PG. This secondary-binding site enhanced the binding of [^19^F]oritavancin to PG as oritavancin is 1000-fold more potent than vancomycin against a broad range of Gram-positive bacteria. The removal of the first amino acid residue of vancomycin, *N*-methylleucine, by Edman degradation leads to the formation of desleucyl-vancomycin, which is devoid of antimicrobial activities due to a damaged primary-binding pocket. On the other hand, the Edman degradation product of oritavancin, desleucyl-oritavancin, still maintains its potent antimicrobial activities despite a damaged primary-binding site. Despite the overwhelming experimental evidence and available NMR data showing the presence of a secondary-binding site in oritavancin, there is still a lack of computational study to compare the binding of oritavancin to PG with that of vancomycin or other glycopeptides. Herein, using explicit solvent MD simulation, COM pulling simulation, and umbrella sampling, we compare the binding of the PG-repeat unit to three different glycopeptides, including oritavancin, chloroeremomycin, and vancomycin, as well as their desleucyl analogs. The free energy of binding to predict the stability of the different (desleucyl)glycopeptide–PG complexes studied was computed through MM/PBSA and umbrella sampling methods. The ΔG_bind_ values obtained from both methods produced the same ranking for the stability of the (desleucyl)glycopeptide–PG complexes (Table 1). The order of stability of the complexes obtained is as follows: Ori–PG > Des-Ori–PG > CEremo–PG > Des-CEremo–PG > Vanco–PG > Des-Vanco–PG. This ranking pattern is also similar to that obtained from the experimental MIC^30^ values, except for the Des-CEremo–PG complex, which showed lower stability than the Vanco–PG complex. Although, this difference in the ranking of Des-CEremo–PG and Vanco–PG complexes is insignificant considering the error estimations.

**Figure 9:**
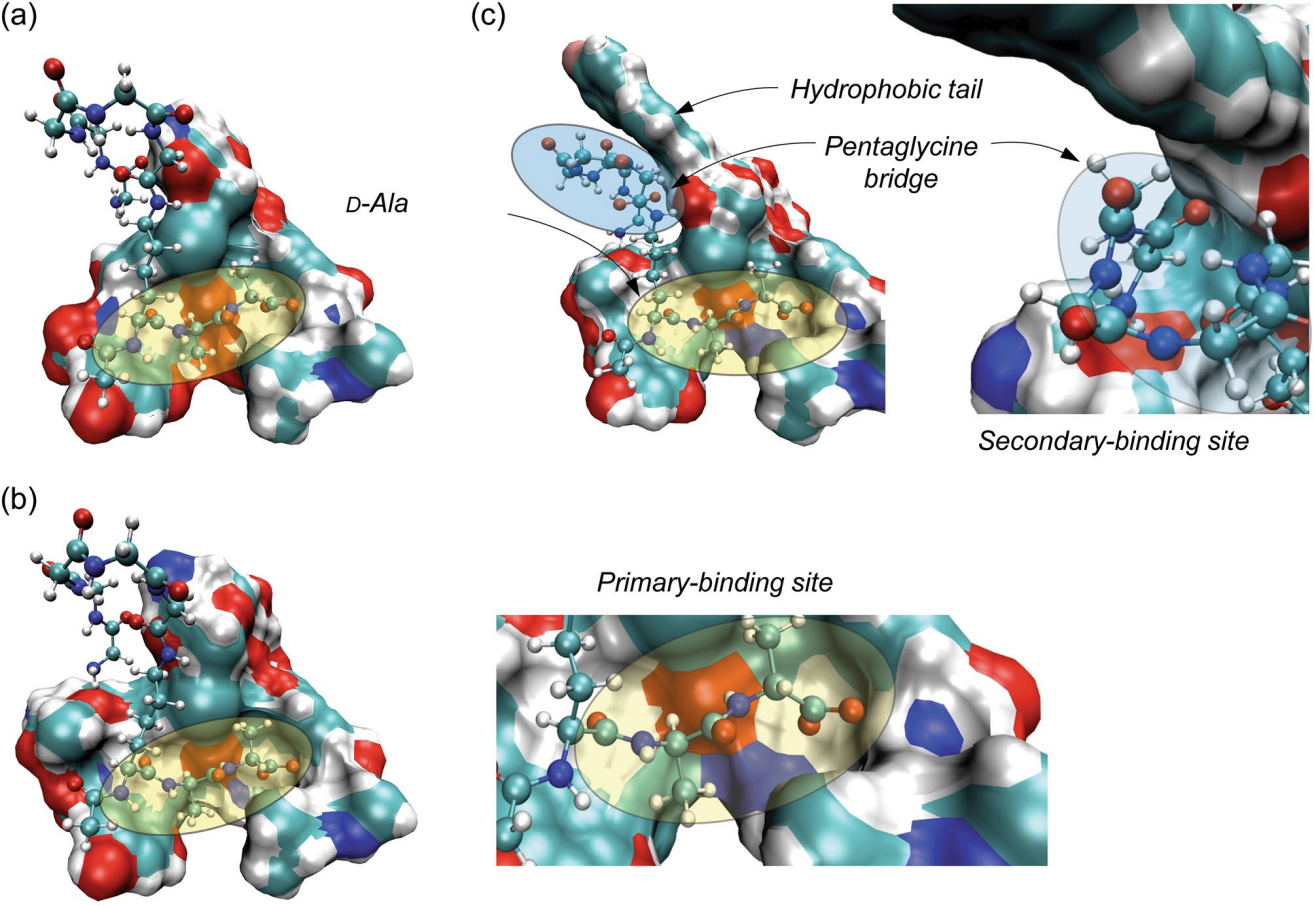
MD simulation model structures of the vancomycin–PG, chloroeremomycin–PG, and [^19^F]oritavancin–PG complexes. (**a**) In the vancomycin–PG complex, the primary-binding site (yellow oval) at the heptapeptide-core of the aglycon is solely responsible for binding to the d-Ala-d-Ala terminus of the PG. (**b**) In the chloroeremomycin–PG complex, the presence of 4-epi-vancosamine attached to the 6th amino acid position enhances the PG binding. Even though chloroeremomycin does not have a secondary-binding site, the difference in the ΔG_bind_ of chloroeremomycin–PG to vancomycin–PG complex (ΔΔG_bind_) is − 16.29 kcal/mol. (**c**) In the oritavancin–PG complex, both primary and secondary-binding sites contribute to the PG binding. The total number of H-bonds formed between the PG and oritavancin is 5.5 which is identical to the number of H-bonds found in the vancomycin–PG complex. But, unlike vancomycin and chloroeremomycin, the secondary-binding site (blue oval) significantly enhances the binding of oritavancin to PG by ΔΔG_bind_ of − 37.83 and − 21.53 kcal/mol in comparison to vancomycin and chloroeremomycin, respectively (Table 2). This enhancement is due to an increase in the ΔG_bind_ by electrostatic and van der Waals interactions between the drug hydrophobic sidechain with the pentaglycine-bridge structure of the bound PG.

The average number of hydrogen bonds, mainly responsible for the strength of interaction between the glycopeptide and PG at the primary-binding pocket, formed between [^19^F]oritavancin and the PG, is less than that formed between chloroeremomycin, vancomycin, and PG. This implies that the enhanced interaction between [^19^F]oritavancin and the PG are not due to an increase in the number of hydrogen bonds but other types of interactions, including hydrophobic effect due to the packing of the pentaglycyl bridge of the PG at the secondary-binding site of [^19^F]oritavancin (Fig. 9). A similar observation applies to the Des-Ori–PG complex with a lower average number of hydrogen bonds than Des-CEremo–PG and Des-Vanco–PG. Typically, five stable hydrogen bonds form at the primary-binding site of [^19^F]oritavancin, chloroeremomycin, and vancomycin. The desleucyl analogs of [^19^F]oritavancin, chloroeremomycin, and vancomycin, formed by removing the first amino acid residue, *N*-methylleucine, show a reduction in the average number of hydrogen bonds formed (Table 1). This removal of *N*-methylleucine, which is not directly involved in the dipeptide binding, is thought to reduce the hydrophobicity of the primary-binding pocket leading to a reduction in the strength of binding of the carboxylate anion of the PG to the three amide NH groups of residues 2–4 of the glycopeptide. The binding of the carboxylate anion of the PG to the neighboring amide hydrogen donors of the glycopeptide is mainly responsible for the strength of the interaction between the glycopeptide and PG at the primary-binding site. The strength of this interaction is estimated to be about 20–30 kJ/mol^47^. The decomposition of the MM/PBSA total binding energy into its van der Waals and electrostatic contribution (Table 2) shows that the increase in the strength of the interaction between [^19^F]oritavancin and PG is a result of a corresponding increase in both the electrostatic and van der Waals interactions, compared to that in the other complexes. The rmsd distribution analysis shows that Ori–PG and Des-Ori–PG complexes are most stable due to a less flexible and narrower rmsd fluctuation pattern. This stability trend is followed by the fluctuation pattern of the CEremo–PG complex. The other complexes, including Vanco–PG, Des-Vanco–PG, and Des-CEremo–PG, show a more flexible rmsd fluctuation pattern in the time evolution of the MD simulation. A 2D rmsd contour plotted by comparing the binding distribution of the Ori–PG complex to that of the other complexes using their rmsd values (Fig. 5) further confirmed that the Des-Ori–PG complex has a binding distribution that is most similar to that of the Ori–PG complex. Both [^19^F]oritavancin and desleucyl[^19^F]oritavancin have a secondary-binding site that enhances their binding to the PG. The binding distribution of the CEremo–PG complex also shows the similarity of binding distribution to that of the Ori–PG complex but with less density of joint conformational distribution.

To further classify the interaction between the (desleucyl)glycopeptide and PG, a force–time curve is plotted after the COM pulling simulation for all the complexes studied. The force–time plot can be used to estimate the interaction between a receptor and ligand when their dissociation pathways are similar. Applying an external force to pull the ligand away from the receptor allows for the calculation of work, which is a path-dependent quantity. Since the dissociation is a path-dependent process, the magnitude of the maximum force applied to pull the PG away from the glycopeptide can be compared to that needed to pull the PG away from the corresponding desleucyl-glycopeptide, as both complexes only differ by *N*-methylleucine. Also, the time it takes to reach this maximum force and the time to reach a major dissociation point can give an insight into what it takes for the PG to escape the attractive pull of the (desleucyl)glycopeptide. The PG requires a comparative higher F_max_ and more time to reach a major dissociation point from the glycopeptide than that needed for the desleucyl-glycopeptide. This implies that the glycopeptides have a stronger attractive pull to the PG than the desleucyl-glycopeptides, mainly caused by the damaged primary-binding pocket of the desleucyl-glycopeptides (Fig. 6). An observation of the force–time plots for all the complexes studied shows that it takes the least amount of time and least F_max_ to pull the PG away from desleucyl-vancomycin.

Overall, our study provides insight into the binding of PG to different glycopeptides, including [^19^F]oritavancin, chloroeremomycin, and vancomycin, as well as their desleucyl analogs. This insight can be helpful in the modification of glycopeptides to increase their antimicrobial activities or the design of novel antibiotics against pathogenic Gram-positive bacteria.

## Conclusions

In this study, we used explicit solvent MD simulation, COM pulling simulations, and umbrella sampling to characterize the binding of PG to three glycopeptide antibiotics and their desleucyl analogs. The glycopeptides simulated include [^19^F]oritavancin, chloroeremomycin, and vancomycin. Their desleucyl analogs are formed by removing the first amino acid residue from the aglycon structure by Edman degradation. [^19^F]oritavancin is known to be 1000-fold more potent than vancomycin against a vast majority of Gram-positive bacteria. This enhanced binding of [^19^F]oritavancin has been shown, from solid-state NMR study, to be due to the presence of a secondary-binding site. As a result of this secondary-binding site, desleucyl [^19^F]oritavancin is known to retain its potency against Gram-positive bacteria, whereas desleucyl-vancomycin is devoid of any antimicrobial activity. To reveal the enhanced binding of [^19^F]oritavancin, MD simulation model structures of [^19^F]oritavancin, chloroeremomycin, and vancomycin complexed with PG referred to as Ori–PG, CEremo–PG, and Vanco–PG respectively, as well as their corresponding desleucyl analogs, Des-Ori–PG, Des-CEremo–PG, and Des-Vanco–PG, were made and used for the simulation studies. Our results show that the ranking of the stability of the (desleucyl)glycopeptide–PG complexes using the free energy of binding values agrees well with the ranking of their stability from the experimental MIC values. Ori–PG complex was shown to be the most stable of all the complexes studied. The average number of hydrogen bonds calculated shows that the enhanced binding of [^19^F]oritavancin over the other complexes is not due to an increase in the electrostatic hydrogen bonding interactions, as [^19^F]oritavancin showed a similar or comparatively lower average number of hydrogen bonding. A decomposition of the binding energy reveals an increase in both electrostatic and van der Waals interactions for [^19^F]oritavancin compared to the other complexes. The rmsd distribution analyses showed that the stabilities of Ori–PG and Des-Ori–PG are higher than that of CEremo–PG, Des-CEremo–PG, Vanco–PG, and Des-Vanco–PG, based on the lower flexibility in their binding distribution. From the COM pulling simulation, the force–time plot was also used to confirm an increase in the attractive pull of the PG by [^19^F]oritavancin and Desleucyl[^19^F]oritavancin when compared to the other complexes. Overall, our MD simulations confirm the presence of a secondary-binding site in Ori–PG and Des-Ori–PG, which is mainly responsible for their enhanced stability of binding compared to the other (desleucyl)glycopeptide–PG complexes modeled. This insight can be useful in the modification of existing glycopeptides to improve their binding efficacy or the design of novel glycopeptides for improved antimicrobial activities against pathogenic Gram-positive bacteria.

## Supplementary Information

Supplementary Information.
