# Supplementary material for: Molecular dynamics simulations of the secondary-binding site in disaccharide-modified glycopeptide antibiotics
Source: Sci Rep. 2022 Apr 30;12:7087. doi: 10.1038/s41598-022-10735-6 (PMC9056522; doi:10.1038/s41598-022-10735-6)
Supplement: Supplementary file 1 — Supplementary Information. [file 41598_2022_10735_MOESM1_ESM.pdf]

Molecular dynamics simulations of the secondary-binding site in disaccharide-modified glycopeptide antibiotics

Olatunde P. Olademehin,[1] Kevin L. Shuford[1], and Sung J. Kim[2]**

[1] Department of Chemistry and Biochemistry, Baylor University, Waco, Texas 76706, U.S.A.

[2] Department of Chemistry, Howard University, Washington, D.C. 20059, U.S.A.

*Corresponding authors

Kevin Shuford: kevin_shuford@baylor.edu Tel: 254-710-2576

Sung Kim: sung.kim@howard.edu Tel: 314-570-3162

Keywords: glycopeptide, vancomycin, chloroeremomycin, oritavancin, *S. aureus*

Supporting Information for publication

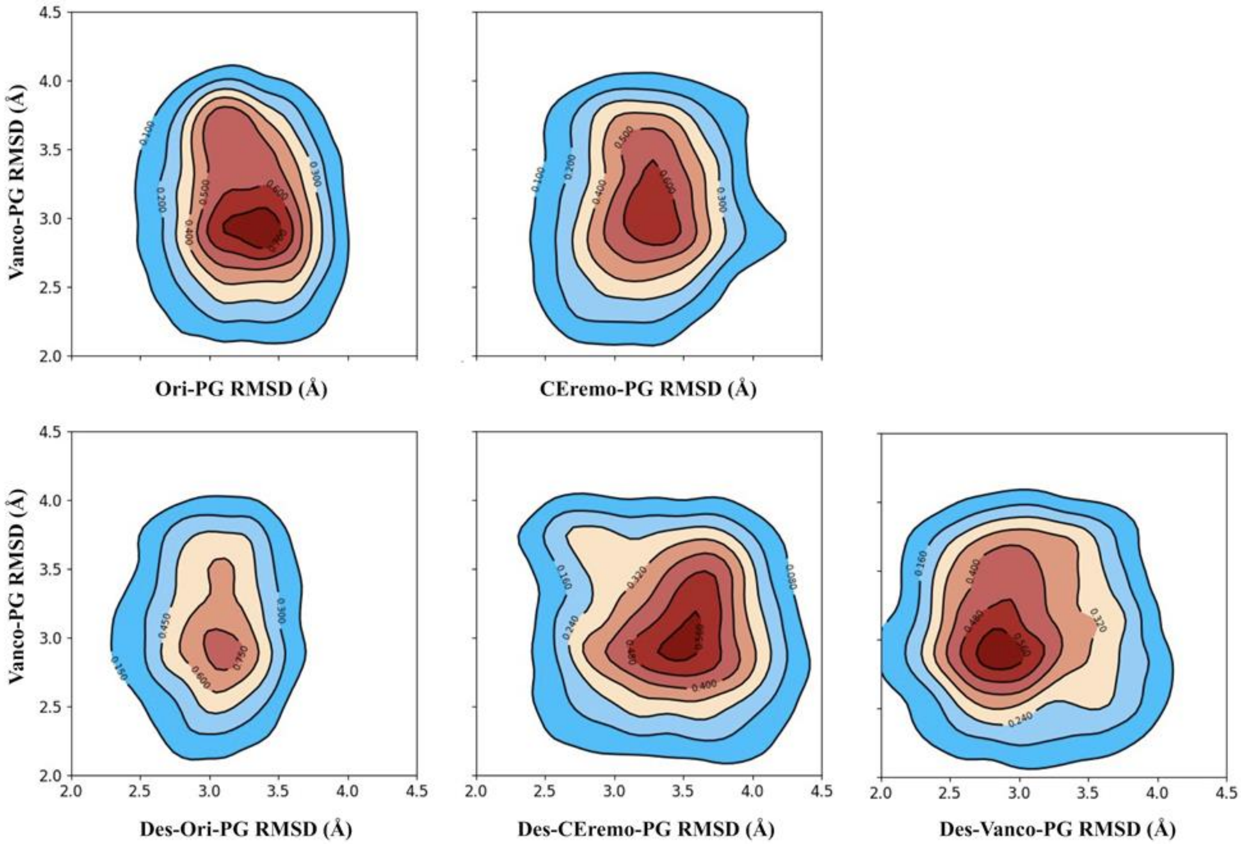

Figure S1. Comparative 2D rmsd (Å) contour plots of the Vanco-PG complex relative to the other complexes. Comparative 2D rmsd contour plots of Vanco-PG vs. Ori-PG (top left), Vanco-PG vs. CEremo-PG (top right), Vanco-PG vs. Des-Ori-PG (bottom left), Vanco-PG vs. Des-CEremo-PG (bottom middle), and Vanco-PG vs. Des-Vanco-PG complexes. The contour lines are the densities of trajectories located within an area. The region of the highest density of trajectories is denoted by the red color, while the blue colors represent regions of lower densities of trajectories.

Table S1. Center-of-mass (COM) pulling simulation data containing time taken to reach major dissociation between PG and (desleucyl)glycopeptide, the magnitude of the maximum force applied in pulling PG away from the (desleucyl)glycopeptide, and the time to reach the maximum force.

Complex	*Major transition time (ps)*	F_{max} *(kJ/mol/nm)*	*Time to reach* F_{max} *(ps)*
Ori-PG	215	645	191
Des-Ori-PG	194	616	141
CEremo-PG	191	719	150
Des-CEremo-PG	186	579	134
Vanco-PG	198	552	127
Des-Vanco-PG	156	437	68

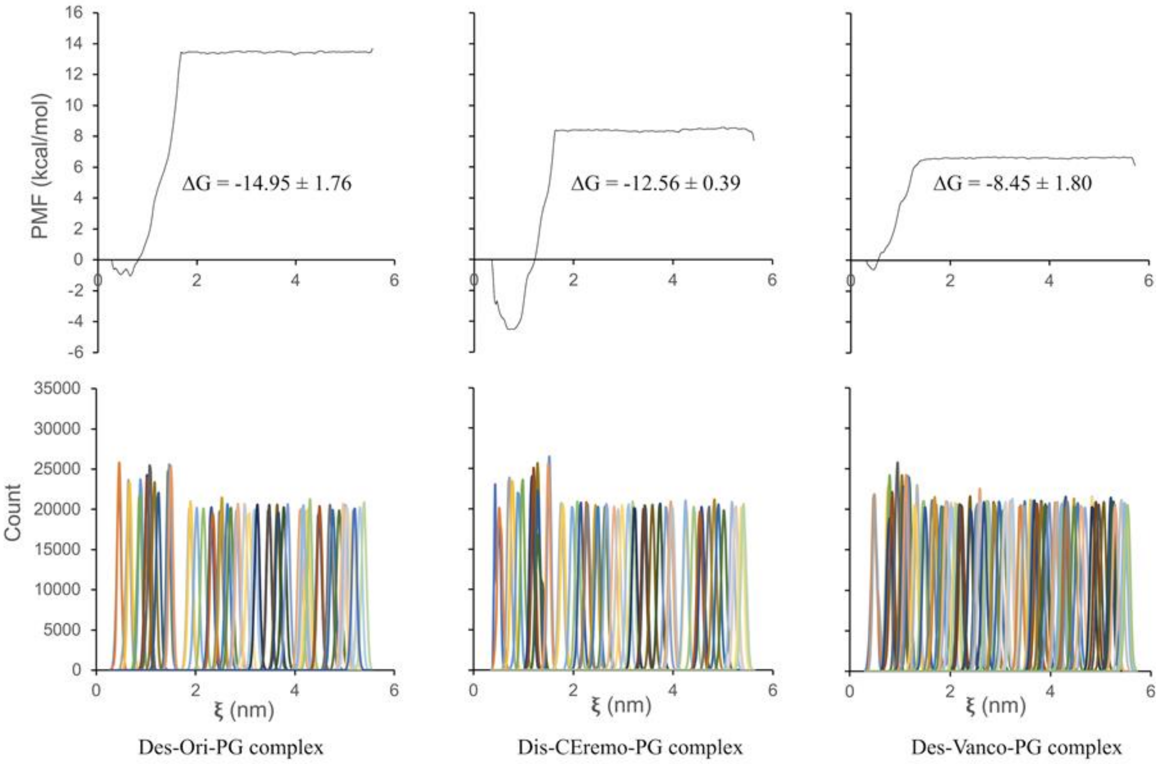

Figure S2. The potential of mean force (PMF) curve and corresponding umbrella histograms for complexes of desleucyl[^{19}F]oritavancin-PG (left), desleucyl-chloroeremomycin-PG (middle), and desleucyl-vancomycin-PG (right). The ΔG values of the complexes obtained from the PMF curve are shown as an inset.

Table S2. Cartesian coordinates for Ori-PG complex

	x	*y*	*z*
C	-1.661539	7.465033	5.120902
C	-0.662690	6.589786	4.381976
O	0.539481	6.796426	4.410927
N	-1.219273	5.556919	3.674659
C	-0.431340	4.736709	2.746723
C	0.514016	3.775857	3.503744
C	1.431474	2.986551	2.566460
C	2.261357	1.915459	3.281676
C	3.211037	1.217834	2.302248
N	3.927489	0.079239	2.873217
C	-1.384038	3.957227	1.824245
O	-1.255446	3.979825	0.596042
N	-2.332114	3.258461	2.476998
C	-3.269869	2.317096	1.874207
C	-4.679945	2.916261	1.750028
C	-3.299407	1.086752	2.807690
O	-2.872057	1.183265	3.961343
N	-3.853389	-0.018380	2.292017
C	-4.116635	-1.207467	3.095341
C	-2.887148	-2.123671	3.190287
C	-5.343578	-1.975024	2.550200
O	-5.748570	-2.914616	3.276361
O	-5.838640	-1.612202	1.449705
N	5.636608	2.436592	-1.791507
C	5.144247	1.289494	-1.032094
C	6.261663	0.546984	-0.293763
O	6.086827	-0.574875	0.197393
N	7.424129	1.223228	-0.230248
C	8.678776	0.652038	0.219668
C	9.439718	1.442871	1.291022
O	10.657834	1.428670	1.298170
N	8.685474	2.104006	2.214637
C	9.315975	2.832853	3.310191
C	9.496320	1.986153	4.585579
O	10.584431	1.801835	5.100364
N	8.334944	1.456490	5.062413
C	8.291674	0.445281	6.105640
C	7.178369	-0.565434	5.803835
O	6.434450	-0.981195	6.668177
N	7.073297	-0.923405	4.472567

C	5.752879	-1.143633	3.917135
C	5.144648	0.186234	3.425032
O	5.752905	1.257144	3.544348
H	-4.370080	-0.873657	4.103915
H	-2.056880	-1.584080	3.650494
H	-3.129042	-2.995629	3.799978
H	-2.579654	-2.458677	2.195473
H	-4.199688	-0.019761	1.335403
H	-2.907118	2.031598	0.888438
H	-4.653677	3.845472	1.179813
H	-5.338296	2.210379	1.242693
H	-5.093287	3.130757	2.739161
H	-2.235924	3.110339	3.478708
H	0.163503	5.390485	2.104276
H	-2.220732	5.559083	3.551543
H	-2.529087	6.900338	5.470711
H	-2.013854	8.254453	4.450267
H	-1.156408	7.923670	5.970022
H	1.110101	4.381580	4.188646
H	-0.084541	3.086545	4.109202
H	0.833438	2.497202	1.789915
H	2.098301	3.685714	2.046516
H	2.846075	2.360135	4.093489
H	1.595465	1.172719	3.733740
H	2.649311	0.842070	1.443504
H	3.950957	1.937560	1.944444
H	3.608117	-0.850358	2.618483
H	5.783032	-1.849668	3.085253
H	5.123825	-1.569664	4.700004
H	7.676752	-0.388283	3.863208
H	9.272925	-0.035616	6.155675
H	8.073008	0.862732	7.091196
H	7.455380	1.659853	4.593595
H	10.311213	3.133186	2.990633
H	8.719696	3.721585	3.534997
H	7.676678	2.041754	2.172035
H	8.468348	-0.347408	0.608052
H	9.376225	0.543159	-0.613811
H	7.399638	2.107075	-0.736405
H	4.437698	1.636270	-0.275169
H	4.610060	0.538724	-1.626346
H	5.863694	2.169571	-2.743234
H	4.938985	3.178393	-1.848068
N	-9.281012	-2.782320	5.983592
C	-8.783729	-2.442612	4.648427
C	-9.240243	-3.473707	3.598984

O	-10.397888	-3.876434	3.595515
C	-9.334935	-1.071140	4.208388
C	-8.887703	0.125330	5.066589
C	-9.702955	1.369124	4.681383
C	-7.385344	0.405330	4.924692
H	-7.089283	1.271246	5.525774
H	-7.130157	0.614991	3.880900
H	-6.779569	-0.442855	5.252297
H	-9.406129	2.237301	5.279671
H	-10.774335	1.204334	4.837362
H	-9.553798	1.614198	3.625205
C	-8.659163	-3.972723	6.557080
N	-8.295271	-3.874187	2.687992
C	-8.690598	-4.691075	1.556652
C	-8.679028	-4.018234	0.174400
O	-9.220683	-4.611422	-0.753851
C	-7.974967	-6.087162	1.531453
O	-7.965284	-6.634007	2.840104
C	-6.587353	-6.071834	0.906653
C	-5.466075	-5.630081	1.614286
C	-6.429750	-6.486718	-0.416811
C	-4.236341	-5.516688	0.980019
C	-5.191622	-6.389827	-1.042510
Cl	-5.023701	-6.901644	-2.715873
C	-4.096911	-5.862405	-0.360635
O	-2.893684	-5.624932	-1.009510
N	-8.118434	-2.782749	0.049145
C	-8.347874	-2.022230	-1.167985
C	-7.125487	-1.497924	-1.939863
O	-7.322196	-1.001008	-3.049464
C	-9.376758	-0.870724	-0.975677
C	-8.857486	0.315465	-0.168613
O	-9.094414	0.459826	1.019459
N	-8.120951	1.215005	-0.886882
H	-7.630636	1.931582	-0.374119
H	-7.820432	1.004143	-1.827254
N	-5.892092	-1.605034	-1.390776
C	-4.734462	-1.171843	-2.157471
C	-4.326416	0.275946	-1.806018
O	-4.131554	0.629414	-0.648968
C	-3.542437	-2.121805	-1.979206
C	-3.762899	-3.426702	-1.556678
C	-2.241382	-1.711577	-2.274244
C	-2.704733	-4.328374	-1.436621
C	-1.182676	-2.603251	-2.125108
C	-1.397079	-3.930330	-1.735856

O	-0.340100	-4.807035	-1.575334
N	-4.145375	1.101445	-2.866106
C	-3.354741	2.322928	-2.760326
C	-2.493047	2.391673	-4.047339
O	-2.905919	1.863002	-5.073798
C	-4.098491	3.654507	-2.657648
C	-3.729593	4.554002	-1.658641
C	-4.131690	5.895721	-1.672426
C	-5.443899	5.399335	-3.661924
C	-4.980813	4.092323	-3.654363
C	-4.995404	6.312931	-2.700217
O	-5.421139	7.600486	-2.799629
N	-1.330357	3.085097	-4.007265
C	-0.729052	3.778207	-2.883037
C	-0.981829	5.298574	-3.046731
O	-1.170041	5.772721	-4.157779
C	0.766251	3.387589	-2.718980
O	1.483071	3.928686	-3.819980
C	0.850110	1.872606	-2.603588
C	0.978196	1.052131	-3.725944
C	0.595465	1.283588	-1.364148
C	0.767141	-0.319111	-3.623455
C	0.394730	-0.088010	-1.258387
Cl	0.040724	-0.782343	0.306791
C	0.429638	-0.894134	-2.402044
O	0.140035	-2.243044	-2.354516
N	-1.068776	5.979255	-1.889803
C	-1.450229	7.387067	-1.847822
C	-0.200957	8.368541	-1.881629
C	-2.443403	7.597387	-0.697252
C	-2.130399	8.486911	0.330339
C	-3.665081	6.875467	-0.632518
C	-2.988906	8.662726	1.412986
O	-2.685100	9.536110	2.428418
C	-4.506807	7.073315	0.481937
O	-5.688174	6.397719	0.609388
C	-4.178868	7.953824	1.509253
O	-0.470492	9.571549	-1.777637
O	0.911577	7.804459	-2.051317
C	0.163345	-5.348341	-2.766913
C	1.363219	-6.245553	-2.457397
O	2.489293	-5.483288	-2.020934
C	1.797452	-6.981861	-3.737175
O	2.844903	-7.903816	-3.465151
C	0.648197	-7.710267	-4.423407
O	1.033841	-8.268178	-5.676093

C	-0.530160	-6.736955	-4.598289
O	-0.862023	-6.136614	-3.348177
C	-1.799995	-7.422810	-5.092188
O	-2.292127	-8.369510	-4.162469
H	0.449441	-4.543101	-3.460315
H	-2.549985	-6.652784	-5.314309
H	-1.567722	-7.964952	-6.010329
C	3.141487	-5.924427	-0.829377
C	4.588076	-5.424352	-0.857744
C	4.756929	-3.922676	-0.538449
N	6.202470	-3.639349	-0.372930
C	4.300913	-3.055046	-1.725003
C	3.922838	-3.633153	0.742077
C	2.468419	-4.121044	0.612582
O	2.470575	-5.538451	0.335771
C	1.665259	-3.947204	1.889058
H	3.121489	-7.018425	-0.805859
H	5.042275	-5.636549	-1.829149
H	5.128516	-6.012146	-0.110790
H	4.422162	-1.994850	-1.494741
H	3.262826	-3.238911	-1.989562
H	4.924697	-3.287569	-2.591661
H	0.658119	-4.340506	1.742611
H	1.593227	-2.891759	2.151691
H	2.133974	-4.490848	2.714696
O	3.910599	-2.250323	1.103185
H	6.363333	-2.641967	-0.476453
C	9.083242	-4.265080	-0.438554
C	8.390622	-3.874531	0.712304
C	9.118349	-3.291903	1.752598
C	10.496234	-3.110817	1.653110
C	11.194816	-3.513189	0.507751
C	10.457902	-4.092684	-0.537081
C	13.502332	-3.550689	1.503434
C	12.664220	-3.333384	0.399607
C	13.259630	-2.944236	-0.809602
C	14.636895	-2.781625	-0.920727
C	15.426698	-3.009097	0.194777
C	14.881114	-3.390027	1.410097
F	16.769621	-2.854264	0.094255
C	6.895166	-4.098010	0.830227
C	2.905190	3.903336	-3.711968
C	3.504381	4.717672	-4.853176
C	3.412763	6.268225	-4.721170
N	4.403385	6.910947	-5.609959
C	2.039530	6.793708	-5.147610

C	3.770819	6.682615	-3.255756
C	3.122752	5.772419	-2.193350
O	3.370587	4.356323	-2.463719
C	3.660989	6.039936	-0.799653
H	3.242125	2.859578	-3.760684
H	3.058748	4.400440	-5.801914
H	4.565662	4.442851	-4.888985
H	2.039630	7.880586	-5.079291
H	1.225146	6.403966	-4.543502
H	1.845288	6.508637	-6.188799
H	3.158583	5.405027	-0.065046
H	3.478295	7.081741	-0.540699
H	4.738373	5.847547	-0.753248
O	3.480705	8.027627	-2.965464
H	-9.095590	-0.123251	6.113390
H	-9.063799	-0.882935	3.164514
H	-10.429501	-1.142650	4.228104
H	-7.691957	-2.419961	4.679583
H	-9.083160	-4.161397	7.547725
H	-8.786268	-4.890513	5.957242
H	-7.586617	-3.799152	6.681961
H	-10.280032	-2.949329	5.888155
H	-7.329924	-3.509318	2.794977
H	-9.746046	-4.912958	1.731575
H	-7.886845	-5.887571	3.449308
H	-8.605681	-6.735771	0.921160
H	-5.556806	-5.307617	2.642230
H	-3.376710	-5.112906	1.500977
H	-7.281448	-6.845390	-0.980707
H	-4.765226	-3.744382	-1.316620
H	-2.039058	-0.700219	-2.596162
H	-5.052665	-1.194496	-3.202923
H	-5.786170	-1.752231	-0.380639
H	-8.806775	-2.732212	-1.859241
H	-7.589814	-2.364733	0.816975
H	-9.679853	-0.537607	-1.970255
H	-10.244049	-1.271523	-0.452473
H	-2.537356	-7.881552	-3.366026
H	-0.240793	-5.959113	-5.327753
H	0.316556	-8.551387	-3.811500
H	1.558527	-7.610505	-6.144986
H	3.605379	-7.383191	-3.187193
H	2.163301	-6.205792	-4.433673
H	1.054601	-6.958875	-1.687440
H	1.969691	-3.596640	-0.206613
H	4.368915	-4.209523	1.565555

H	4.651388	-1.770918	0.693512
H	6.546007	-3.620286	1.758510
H	6.712001	-5.171096	0.955575
H	8.602528	-2.962406	2.649590
H	8.526041	-4.699498	-1.260115
H	11.027568	-2.617820	2.459264
H	10.973859	-4.433615	-1.428213
H	15.531001	-3.561398	2.259313
H	13.071421	-3.868700	2.445279
H	12.633906	-2.739062	-1.669987
H	15.098453	-2.468826	-1.849217
H	-4.247627	0.752230	-3.812343
H	0.487011	1.902198	-0.481012
H	1.179731	1.497839	-4.691531
H	0.802116	-0.958306	-4.497523
H	1.121889	3.838365	-1.789181
H	2.046393	5.952079	-2.200624
H	4.863062	6.571024	-3.161978
H	2.535658	8.104924	-2.687425
H	5.333889	6.545666	-5.421933
H	4.186850	6.685253	-6.577699
H	-0.970646	3.350671	-4.915607
H	-1.206205	3.448274	-1.964149
H	-0.905477	5.497655	-1.013609
H	-1.964789	7.578819	-2.794608
H	-4.851095	8.090605	2.346188
H	-1.236566	9.091597	0.234836
H	-1.849348	9.964990	2.211770
H	-5.779728	5.806319	-0.150657
H	-4.966779	8.117362	-2.118192
H	-6.113644	5.752718	-4.436606
H	-5.275450	3.417498	-4.450312
H	-3.062112	4.233286	-0.867949
H	-2.741094	2.215837	-1.866728

Table S3. Cartesian coordinates for Des-Ori-PG complex

	x	*y*	*z*
C	-2.144910	7.475828	5.278923
C	-1.167289	6.630927	4.478818
O	0.014171	6.916654	4.372678
N	-1.718987	5.529230	3.879829
C	-0.973698	4.715787	2.911583

C	0.108547	3.858472	3.607120
C	0.979260	3.083787	2.614671
C	1.950397	2.106953	3.285615
C	2.842389	1.423582	2.243882
N	3.691061	0.364722	2.786356
C	-1.958065	3.829813	2.129110
O	-1.956594	3.798000	0.894397
N	-2.783751	3.105009	2.907641
C	-3.709525	2.075005	2.448358
C	-5.163424	2.573911	2.446136
C	-3.557961	0.894621	3.433107
O	-3.023636	1.077548	4.530328
N	-4.082580	-0.269139	3.027435
C	-4.179032	-1.430278	3.905505
C	-2.885095	-2.258393	3.911550
C	-5.398121	-2.302967	3.526102
O	-5.660546	-3.228642	4.331348
O	-6.026436	-2.029832	2.468542
N	4.748901	2.587154	-2.130922
C	4.417685	1.450691	-1.274353
C	5.653483	0.821224	-0.625010
O	5.608398	-0.283098	-0.069303
N	6.766142	1.574122	-0.713348
C	8.096871	1.109601	-0.372464
C	8.905247	2.001995	0.577488
O	10.115794	2.067565	0.457747
N	8.204098	2.658884	1.544925
C	8.889826	3.482243	2.535302
C	9.257465	2.715317	3.820878
O	10.402399	2.628803	4.226592
N	8.190416	2.136158	4.439497
C	8.324128	1.178866	5.525063
C	7.259526	0.083681	5.386244
O	6.638068	-0.334985	6.341163
N	7.045328	-0.347880	4.090354
C	5.694083	-0.681734	3.686391
C	4.947402	0.578819	3.202520
O	5.488083	1.691773	3.210227
H	-4.351650	-1.062628	4.919370
H	-2.052296	-1.643066	4.258123
H	-3.002216	-3.111987	4.581032
H	-2.657361	-2.623011	2.905753
H	-4.523155	-0.341919	2.113306
H	-3.429507	1.763786	1.443584
H	-5.260293	3.472530	1.835916
H	-5.818745	1.801658	2.042077

H	-5.488331	2.811247	3.462675
H	-2.576328	3.014808	3.899335
H	-0.494354	5.373269	2.182237
H	-2.725446	5.459858	3.862210
H	-2.930799	6.874413	5.742136
H	-2.617864	8.205345	4.614709
H	-1.589655	8.009154	6.049485
H	0.727102	4.535920	4.198527
H	-0.375744	3.163445	4.301671
H	0.341320	2.517507	1.927290
H	1.539282	3.797445	1.997503
H	2.581827	2.629646	4.011397
H	1.387472	1.346463	3.837164
H	2.224187	0.968666	1.466029
H	3.489949	2.170773	1.779251
H	3.413427	-0.595498	2.607650
H	5.689112	-1.425856	2.887843
H	5.179072	-1.107311	4.548796
H	7.544888	0.193606	3.398209
H	9.336666	0.766099	5.493282
H	8.177828	1.630960	6.508733
H	7.255766	2.257585	4.056791
H	9.824022	3.830066	2.100432
H	8.258620	4.340557	2.781891
H	7.203037	2.528950	1.610906
H	7.997465	0.119706	0.079467
H	8.712104	1.003975	-1.268739
H	6.628576	2.427496	-1.252613
H	3.768989	1.788896	-0.463667
H	3.879912	0.637355	-1.775782
H	4.896446	2.287249	-3.088492
H	3.998885	3.278074	-2.146801
N	-8.180258	-4.380872	4.055561
C	-8.630065	-5.278476	3.008900
C	-8.805908	-4.677767	1.604729
O	-9.396006	-5.351602	0.765299
C	-7.824625	-6.624438	2.970493
O	-7.643984	-7.101958	4.293764
C	-6.512031	-6.550665	2.203853
C	-5.358493	-6.001249	2.770111
C	-6.460777	-7.021433	0.890737
C	-4.210406	-5.840517	2.006265
C	-5.302335	-6.876162	0.135252
Cl	-5.269535	-7.460723	-1.522309
C	-4.183777	-6.244417	0.674924
O	-3.072170	-5.962503	-0.106129

N	-8.348861	-3.416396	1.367182
C	-8.753274	-2.735579	0.148422
C	-7.655226	-2.172849	-0.769481
O	-7.997782	-1.747108	-1.873218
C	-9.835493	-1.645234	0.396414
C	-9.321448	-0.388079	1.091654
O	-9.446198	-0.198987	2.290475
N	-8.726403	0.519684	0.261320
H	-8.238035	1.291925	0.687884
H	-8.508767	0.281109	-0.695127
N	-6.368047	-2.171203	-0.348293
C	-5.327352	-1.703229	-1.250256
C	-4.988220	-0.215880	-1.007568
O	-4.702270	0.208147	0.106033
C	-4.059776	-2.563172	-1.156023
C	-4.144274	-3.856380	-0.655661
C	-2.827309	-2.084657	-1.603376
C	-3.018824	-4.679892	-0.607380
C	-1.699074	-2.896684	-1.526702
C	-1.779379	-4.213303	-1.059057
O	-0.652752	-5.009944	-0.971549
N	-4.973909	0.564453	-2.116120
C	-4.264165	1.838653	-2.147580
C	-3.544381	1.897586	-3.519470
O	-4.021158	1.291422	-4.472792
C	-5.085132	3.122453	-2.026411
C	-4.680761	4.093902	-1.112101
C	-5.175202	5.404117	-1.142840
C	-6.644385	4.722367	-2.960286
C	-6.092483	3.450394	-2.943642
C	-6.165795	5.711149	-2.091986
O	-6.688582	6.961475	-2.202937
N	-2.435081	2.666422	-3.631878
C	-1.772915	3.453729	-2.608383
C	-2.146895	4.944053	-2.811558
O	-2.479570	5.347583	-3.916618
C	-0.245007	3.170243	-2.584649
O	0.316775	3.699966	-3.777382
C	-0.043933	1.671813	-2.412024
C	0.026844	0.805192	-3.504373
C	-0.129667	1.131511	-1.127999
C	-0.076127	-0.569862	-3.320041
C	-0.222040	-0.243057	-0.941480
Cl	-0.365964	-0.878154	0.681215
C	-0.246897	-1.102535	-2.045874
O	-0.434955	-2.463256	-1.908911

N	-2.163545	5.675806	-1.683006
C	-2.636475	7.056080	-1.663412
C	-1.468929	8.113846	-1.870975
C	-3.520128	7.259723	-0.425621
C	-3.167626	8.219053	0.523376
C	-4.675340	6.463996	-0.201655
C	-3.922110	8.393614	1.681113
O	-3.578839	9.335640	2.619703
C	-5.411488	6.663232	0.984957
O	-6.523582	5.919500	1.264975
C	-5.043562	7.614538	1.932515
O	-1.810156	9.300476	-1.792458
O	-0.342564	7.615514	-2.131130
C	-0.236061	-5.577435	-2.184253
C	1.048918	-6.377597	-1.962764
O	2.157257	-5.522302	-1.680547
C	1.401645	-7.148433	-3.247262
O	2.533295	-7.985097	-3.045982
C	0.242599	-7.984249	-3.776698
O	0.537354	-8.579082	-5.037056
C	-1.012706	-7.099972	-3.870029
O	-1.257324	-6.459482	-2.619726
C	-2.274888	-7.891418	-4.197633
O	-2.602646	-8.819460	-3.180714
H	-0.078890	-4.791725	-2.938590
H	-3.095639	-7.184235	-4.373765
H	-2.099511	-8.463447	-5.110254
C	2.956228	-5.858599	-0.545506
C	4.353868	-5.267447	-0.747101
C	4.448594	-3.743621	-0.513708
N	5.880044	-3.358642	-0.513010
C	3.814929	-2.969260	-1.683100
C	3.730441	-3.444048	0.833062
C	2.308167	-4.031628	0.878098
O	2.381550	-5.458164	0.665307
C	1.628404	-3.845532	2.222705
H	3.015422	-6.948945	-0.471843
H	4.720958	-5.498925	-1.750403
H	5.007124	-5.779877	-0.035631
H	3.884318	-1.893047	-1.513730
H	2.770812	-3.233631	-1.829217
H	4.362357	-3.204599	-2.599182
H	0.641651	-4.310574	2.199926
H	1.509602	-2.784992	2.444672
H	2.215368	-4.314619	3.017910
O	3.658023	-2.048322	1.132185

H	5.959297	-2.359489	-0.676644
C	8.776036	-3.797903	-0.852092
C	8.178046	-3.395055	0.346593
C	8.964899	-2.713900	1.278335
C	10.309498	-2.448750	1.027325
C	10.914791	-2.862712	-0.166000
C	10.118141	-3.541574	-1.101428
C	13.308313	-2.698917	0.583430
C	12.349423	-2.593318	-0.435131
C	12.790394	-2.228457	-1.715973
C	14.134492	-1.982353	-1.977667
C	15.047338	-2.100635	-0.942001
C	14.655857	-2.453748	0.339336
F	16.358976	-1.863978	-1.189224
C	6.721612	-3.709170	0.630090
C	1.740779	3.772864	-3.817767
C	2.162606	4.565173	-5.050178
C	1.976429	6.111177	-4.977760
N	2.824326	6.770849	-5.992784
C	0.533602	6.523621	-5.280897
C	2.451332	6.622293	-3.577550
C	1.979726	5.727246	-2.414093
O	2.297476	4.318351	-2.646251
C	2.636454	6.100071	-1.097519
H	2.143339	2.752151	-3.855435
H	1.646385	4.171556	-5.932170
H	3.231418	4.358606	-5.184703
H	0.464473	7.610264	-5.260921
H	-0.185998	6.113006	-4.578302
H	0.255224	6.173712	-6.282466
H	2.256866	5.472118	-0.287114
H	2.408417	7.139621	-0.867027
H	3.723808	5.980900	-1.155679
O	2.098627	7.958612	-3.318042
H	-7.529977	-3.723707	3.674420
H	-8.965323	-3.891232	4.434935
H	-9.644177	-5.559426	3.302914
H	-7.556663	-6.321868	4.857930
H	-8.466974	-7.343072	2.458726
H	-5.366879	-5.633317	3.786693
H	-3.332791	-5.355518	2.416108
H	-7.338035	-7.463152	0.435416
H	-5.092353	-4.226012	-0.298270
H	-2.730039	-1.080025	-1.988948
H	-5.747564	-1.799914	-2.254651
H	-6.150177	-2.259418	0.650714

H	-9.229030	-3.508316	-0.459059
H	-7.775693	-2.926172	2.056311
H	-10.260482	-1.383788	-0.574693
H	-10.615026	-2.074445	1.024652
H	-2.799425	-8.308487	-2.385260
H	-0.854026	-6.343194	-4.659330
H	0.034479	-8.812844	-3.096982
H	0.964404	-7.913426	-5.586800
H	3.279680	-7.402523	-2.872341
H	1.639691	-6.386756	-4.011735
H	0.870720	-7.069263	-1.134060
H	1.693420	-3.583341	0.093283
H	4.297013	-3.947333	1.629932
H	4.318025	-1.543220	0.626518
H	6.435784	-3.208435	1.567858
H	6.627746	-4.784107	0.821078
H	8.520892	-2.373339	2.208985
H	8.170206	-4.309078	-1.590891
H	10.883992	-1.881669	1.750942
H	10.563678	-3.893222	-2.025766
H	15.398918	-2.538833	1.122605
H	12.998487	-2.995819	1.578288
H	12.067811	-2.108682	-2.514357
H	14.476451	-1.687966	-2.962234
H	-5.146874	0.161461	-3.030150
H	-0.191077	1.786025	-0.266482
H	0.097687	1.213198	-4.504444
H	-0.085274	-1.249214	-4.163952
H	0.170628	3.690115	-1.717940
H	0.898250	5.835797	-2.316602
H	3.552575	6.587030	-3.593758
H	1.183568	7.988273	-2.946314
H	3.792397	6.477041	-5.887250
H	2.527114	6.482411	-6.921662
H	-2.188840	2.908140	-4.583689
H	-2.130123	3.140760	-1.630968
H	-1.878888	5.251234	-0.808346
H	-3.256564	7.165310	-2.558679
H	-5.635370	7.749789	2.828355
H	-2.332665	8.875019	0.308313
H	-2.801483	9.806434	2.298102
H	-6.650079	5.285357	0.545484
H	-6.204797	7.540971	-1.596228
H	-7.412347	4.991351	-3.675444
H	-6.418310	2.718050	-3.673877
H	-3.915711	3.858143	-0.382238

H	-3.557071	1.817533	-1.319257

Table S4. Cartesian coordinates for CEremo-PG complex

	x	*y*	*z*
C	2.616910	6.853786	4.982632
C	3.114222	5.594022	4.293106
O	4.276137	5.226634	4.354195
N	2.157440	4.909300	3.591237
C	2.496038	3.789015	2.705256
C	2.880682	2.526834	3.510838
C	3.348654	1.374229	2.618797
C	3.580814	0.065805	3.381429
C	4.120268	-1.021574	2.446310
N	4.223171	-2.342592	3.062777
C	1.307729	3.504021	1.770969
O	1.453494	3.424804	0.547038
N	0.133441	3.340198	2.409262
C	-1.121531	2.915099	1.798325
C	-2.095991	4.090212	1.618688
C	-1.729437	1.866916	2.756329
O	-1.325532	1.794261	3.920133
N	-2.720436	1.123122	2.247582
C	-3.514643	0.214574	3.067540
C	-2.845764	-1.160084	3.219191
C	-4.947489	0.078414	2.501744
O	-5.751534	-0.546092	3.235000
O	-5.201285	0.591702	1.379274
N	6.904868	-1.187042	-1.596736
C	5.927285	-1.954586	-0.828511
C	6.565394	-3.102505	-0.041001
O	5.885979	-4.002177	0.468266
N	7.907327	-3.033606	0.043474
C	8.751017	-4.101864	0.543309
C	9.771711	-3.713896	1.620302
O	10.846644	-4.285387	1.664765
N	9.389977	-2.750448	2.506358
C	10.265938	-2.356848	3.604884
C	10.014844	-3.149550	4.902811
O	10.887382	-3.795765	5.454214
N	8.731933	-3.071099	5.355242
C	8.210673	-3.914827	6.417856

C	6.762753	-4.311055	6.103833
O	5.896180	-4.310391	6.953780
N	6.527882	-4.624279	4.777867
C	5.263813	-4.231936	4.187353
C	5.343565	-2.788189	3.648936
O	6.373865	-2.112682	3.763973
H	-3.603641	0.660330	4.060650
H	-1.868458	-1.047029	3.692885
H	-3.471892	-1.803222	3.839581
H	-2.709561	-1.631259	2.241500
H	-3.012044	1.249493	1.281130
H	-0.913663	2.462812	0.830303
H	-1.635684	4.884448	1.029981
H	-2.996276	3.749064	1.106645
H	-2.381475	4.502793	2.590037
H	0.133472	3.197372	3.416345
H	3.335826	4.075367	2.067480
H	1.271349	5.366857	3.437725
H	1.580983	6.762103	5.317797
H	2.678541	7.694389	4.285030
H	3.261596	7.056953	5.836806
H	3.676574	2.813459	4.200556
H	2.021899	2.209425	4.112291
H	2.606106	1.188817	1.834976
H	4.271107	1.671851	2.104666
H	4.290393	0.218824	4.201036
H	2.640331	-0.273160	3.828678
H	3.463592	-1.125503	1.579044
H	5.114276	-0.733931	2.095994
H	3.516673	-3.029674	2.818201
H	4.980393	-4.899882	3.371913
H	4.495916	-4.295783	4.959592
H	7.320202	-4.446140	4.175931
H	8.860018	-4.790538	6.507863
H	8.191401	-3.411531	7.387249
H	8.052528	-2.502193	4.855708
H	11.293198	-2.557492	3.309429
H	10.141147	-1.286743	3.792591
H	8.466370	-2.344075	2.434447
H	8.098044	-4.879886	0.946200
H	9.334628	-4.545978	-0.266097
H	8.300606	-2.254248	-0.481800
H	5.446272	-1.297591	-0.100922
H	5.118113	-2.395428	-1.422687
H	7.000182	-1.559539	-2.535253
H	6.627406	-0.210092	-1.690171

N	-8.873956	1.281493	5.830220
C	-8.253208	1.311081	4.503888
C	-9.114418	0.570786	3.463131
O	-10.327376	0.744480	3.433014
C	-8.104597	2.767297	4.018351
C	-7.172308	3.652409	4.864213
C	-7.317785	5.118264	4.428151
C	-5.707180	3.206766	4.762139
H	-5.056631	3.859362	5.353465
H	-5.366203	3.241567	3.722378
H	-5.564853	2.186408	5.125894
H	-6.665469	5.772424	5.016660
H	-8.347523	5.468835	4.554954
H	-7.054461	5.232767	3.372040
C	-8.878911	-0.042016	6.447426
N	-8.443733	-0.248374	2.589965
C	-9.150733	-0.829259	1.464690
C	-8.807310	-0.282506	0.069487
O	-9.544911	-0.590961	-0.862024
C	-9.156634	-2.398049	1.490826
O	-9.421998	-2.845106	2.810456
C	-7.906720	-3.041836	0.908300
C	-6.720267	-3.141108	1.640166
C	-7.934655	-3.525901	-0.400679
C	-5.565295	-3.625673	1.041305
C	-6.779964	-4.028689	-0.990374
Cl	-6.837256	-4.615018	-2.646644
C	-5.577275	-4.040551	-0.286869
O	-4.388549	-4.403266	-0.903763
N	-7.739457	0.552977	-0.064814
C	-7.572577	1.293610	-1.304190
C	-6.232864	1.172728	-2.049495
O	-6.159951	1.667826	-3.174838
C	-7.960131	2.794514	-1.167672
C	-6.967721	3.635756	-0.370505
O	-7.132238	3.911615	0.806493
N	-5.887840	4.072782	-1.084951
H	-5.131934	4.500733	-0.572817
H	-5.701704	3.716812	-2.011078
N	-5.196427	0.529500	-1.461033
C	-3.956186	0.357617	-2.201197
C	-2.934418	1.467234	-1.868250
O	-2.618884	1.729536	-0.713559
C	-3.337526	-1.027087	-1.966708
C	-4.140408	-2.070573	-1.523531
C	-1.988632	-1.269730	-2.230693

C	-3.617400	-3.352870	-1.352300
C	-1.461051	-2.542445	-2.030599
C	-2.268175	-3.609497	-1.620021
O	-1.735443	-4.867756	-1.408919
N	-2.375905	2.082294	-2.939593
C	-1.114221	2.807329	-2.835730
C	-0.295295	2.430661	-4.097129
O	-0.887196	2.117156	-5.124233
C	-1.164277	4.334294	-2.783929
C	-0.440487	4.996312	-1.793449
C	-0.180513	6.371643	-1.847863
C	-1.539486	6.468033	-3.865360
C	-1.729246	5.095318	-3.816047
C	-0.737895	7.104813	-2.910033
O	-0.522330	8.440099	-3.049587
N	1.055138	2.513941	-4.036435
C	1.888254	2.890154	-2.909386
C	2.365520	4.350643	-3.112852
O	2.435536	4.821578	-4.238900
C	3.033565	1.862383	-2.691726
O	3.937776	1.977677	-3.781647
C	2.409622	0.482625	-2.541776
C	2.165565	-0.341396	-3.642091
C	1.891366	0.117184	-1.298449
C	1.346061	-1.458594	-3.517042
C	1.080806	-1.004959	-1.169810
Cl	0.420292	-1.407619	0.398242
C	0.760979	-1.774169	-2.294547
O	-0.117055	-2.837287	-2.227282
N	2.581248	5.032801	-1.973778
C	2.888971	6.459163	-1.973236
C	4.449969	6.756021	-1.989843
C	2.083939	7.139472	-0.858093
C	2.753023	7.819230	0.159464
C	0.666221	7.061402	-0.815240
C	2.052875	8.404865	1.211554
O	2.706582	9.074117	2.217023
C	-0.009449	7.659939	0.268680
O	-1.371162	7.606550	0.374451
C	0.668757	8.324829	1.286607
O	4.761852	7.951214	-1.919594
O	5.181436	5.738995	-2.113657
C	-1.516690	-5.618482	-2.572915
C	-0.869077	-6.955653	-2.208143
O	0.473648	-6.781454	-1.753904
C	-0.799895	-7.850630	-3.458251

O	-0.298348	-9.141035	-3.135054
C	-2.143328	-7.992314	-4.163510
O	-2.035750	-8.705714	-5.391799
C	-2.739177	-6.593042	-4.394698
O	-2.779453	-5.866741	-3.168245
C	-4.173434	-6.635298	-4.912321
O	-5.061634	-7.219445	-3.978144
H	-0.880538	-5.057610	-3.274202
H	-4.481561	-5.614679	-5.173392
H	-4.200562	-7.253335	-5.811290
C	0.829392	-7.433414	-0.534001
C	2.344207	-7.654348	-0.528978
C	3.178917	-6.388152	-0.236577
N	4.589819	-6.794685	-0.033243
C	3.193326	-5.447422	-1.454553
C	2.549302	-5.706284	1.011589
C	1.035861	-5.476113	0.848317
O	0.390996	-6.744593	0.601783
C	0.380603	-4.911283	2.095827
H	0.308338	-8.394749	-0.487935
H	2.666720	-8.083060	-1.481419
H	2.540981	-8.399789	0.246337
H	3.784364	-4.554254	-1.243219
H	2.191639	-5.142851	-1.746583
H	3.655265	-5.968587	-2.296734
H	-0.691919	-4.802990	1.927204
H	0.797281	-3.932555	2.333736
H	0.532629	-5.582026	2.946657
O	3.167865	-4.461112	1.342788
H	5.109738	-6.022740	0.332523
H	4.627194	-7.557524	0.612265
C	5.186981	1.305957	-3.629953
C	6.113044	1.716515	-4.769417
C	6.742178	3.139523	-4.673128
N	7.932555	3.226324	-5.544815
C	5.771778	4.222402	-5.152138
C	7.225298	3.391114	-3.206615
C	6.213131	2.915300	-2.145355
O	5.786900	1.535428	-2.378012
C	6.789959	2.951439	-0.741923
H	5.007194	0.223026	-3.646289
H	5.587908	1.608317	-5.724327
H	6.929660	0.984218	-4.767128
H	6.270307	5.189559	-5.106883
H	4.859090	4.269981	-4.564939
H	5.486236	4.024338	-6.192477

H	6.039330	2.642430	-0.009641
H	7.102172	3.968730	-0.510765
H	7.657347	2.287630	-0.658733
O	7.580969	4.727935	-2.953827
H	-7.489230	3.561442	5.909243
H	-7.759320	2.775725	2.979447
H	-9.109691	3.206862	4.006305
H	-7.273932	0.831088	4.567722
H	-9.359213	0.017559	7.428502
H	-9.403309	-0.818177	5.863576
H	-7.848949	-0.376121	6.601139
H	-9.836125	1.588641	5.708025
H	-7.420709	-0.364021	2.718485
H	-10.192896	-0.536068	1.612117
H	-9.019742	-2.198434	3.405815
H	-10.004276	-2.704409	0.875522
H	-6.670339	-2.779476	2.657817
H	-4.625396	-3.644679	1.579477
H	-8.846034	-3.471893	-0.982474
H	-5.180565	-1.884682	-1.307370
H	-1.338526	-0.475233	-2.567663
H	-4.231093	0.449294	-3.255122
H	-5.187475	0.383304	-0.445253
H	-8.294478	0.851245	-1.993880
H	-7.091185	0.706634	0.709700
H	-8.058960	3.196788	-2.177834
H	-8.923483	2.853909	-0.662695
H	-5.068817	-6.647556	-3.200036
H	-2.112093	-6.059179	-5.131342
H	-2.835006	-8.566806	-3.544286
H	-1.259459	-8.378012	-5.858355
H	0.611416	-9.018757	-2.845203
H	-0.106285	-7.352366	-4.159591
H	-1.484294	-7.422070	-1.432985
H	0.848230	-4.808465	0.003421
H	2.666221	-6.395763	1.860266
H	4.053068	-4.388932	0.945755
H	-2.610893	1.788170	-3.880891
H	2.064190	0.745131	-0.432318
H	2.566061	-0.069837	-4.610331
H	1.098375	-2.070793	-4.375909
H	3.540508	2.129833	-1.761305
H	5.340127	3.568619	-2.189139
H	8.142202	2.793773	-3.077345
H	6.772580	5.239453	-2.706336
H	8.587606	2.481076	-5.320903

H	7.653245	3.093616	-6.513691
H	1.512289	2.554846	-4.938847
H	1.297058	2.846361	-1.998641
H	2.489738	4.558986	-1.083022
H	2.536571	6.834558	-2.939071
H	0.120307	8.782253	2.099641
H	3.826283	7.942688	0.078382
H	3.649547	9.064178	2.016838
H	-1.711193	7.098599	-0.375328
H	0.106941	8.712781	-2.365757
H	-1.958349	7.063800	-4.667348
H	-2.287326	4.605338	-4.606111
H	-0.008894	4.431112	-0.976222
H	-0.634005	2.459875	-1.921828

Table S5. Cartesian coordinates for Des-CEremo-PG complex

	x	*y*	*z*
C	3.956268	6.065362	5.362953
C	4.133964	4.811579	4.522584
O	5.214648	4.263022	4.381303
N	2.987515	4.351809	3.929994
C	3.006430	3.277942	2.929445
C	3.281765	1.902966	3.580326
C	3.420663	0.777637	2.551941
C	3.533440	-0.615058	3.180559
C	3.747067	-1.683483	2.103210
N	3.711249	-3.055017	2.606520
C	1.668873	3.268703	2.169807
O	1.627541	3.279690	0.935424
N	0.584140	3.232941	2.966853
C	-0.797035	3.066829	2.526773
C	-1.575529	4.391275	2.576998
C	-1.433173	2.043591	3.493377
O	-0.889300	1.802788	4.574614
N	-2.596075	1.513453	3.092449
C	-3.409765	0.669269	3.960675

C	-2.966918	-0.801094	3.915921
C	-4.909691	0.811013	3.611617
O	-5.697309	0.255627	4.414963
O	-5.227271	1.457928	2.577954
N	5.874315	-1.912656	-2.318316
C	4.898166	-2.584774	-1.463710
C	5.437549	-3.885356	-0.861526
O	4.694154	-4.710409	-0.316529
N	6.770488	-4.035115	-0.977774
C	7.483865	-5.263244	-0.685175
C	8.695002	-5.139548	0.247614
O	9.654185	-5.873719	0.089437
N	8.607644	-4.213189	1.244246
C	9.681477	-4.063290	2.220667
C	9.483781	-4.922074	3.485360
O	10.303413	-5.744349	3.852824
N	8.307900	-4.685622	4.131968
C	7.805234	-5.530875	5.202300
C	6.281917	-5.665519	5.086296
O	5.554574	-5.606936	6.056296
N	5.815597	-5.815850	3.793468
C	4.565761	-5.178467	3.429926
C	4.811316	-3.721913	2.984045
O	5.946324	-3.229152	2.986175
H	-3.283547	1.030952	4.983485
H	-1.928255	-0.885933	4.241919
H	-3.599738	-1.392322	4.579649
H	-3.049196	-1.196564	2.899422
H	-2.993874	1.771519	2.192341
H	-0.804855	2.677848	1.510176
H	-1.074736	5.154140	1.979911
H	-2.582695	4.243248	2.186115
H	-1.649667	4.753038	3.606015
H	0.700272	3.000573	3.950283
H	3.785384	3.486460	2.192051
H	2.178306	4.954354	3.944241
H	2.976515	6.106578	5.844898
H	4.060632	6.946098	4.722270
H	4.738400	6.086432	6.120802
H	4.202607	1.997479	4.158621
H	2.474000	1.670188	4.282702
H	2.555797	0.782910	1.879548
H	4.299386	0.973031	1.924649
H	4.365665	-0.650299	3.891074
H	2.620868	-0.842190	3.741862
H	2.968090	-1.603885	1.340954

H	4.716734	-1.524039	1.625890
H	2.872277	-3.597968	2.426609
H	4.063472	-5.716654	2.624021
H	3.913065	-5.191667	4.303944
H	6.535028	-5.709606	3.091354
H	8.304291	-6.501855	5.133313
H	8.005869	-5.121339	6.194986
H	7.670974	-3.974307	3.781060
H	10.609170	-4.394271	1.759523
H	9.765642	-3.008483	2.496766
H	7.764574	-3.662563	1.341218
H	6.771920	-5.963084	-0.240907
H	7.866178	-5.717409	-1.601989
H	7.212192	-3.282191	-1.503115
H	4.640373	-1.929822	-0.628856
H	3.951738	-2.837599	-1.956154
H	5.774167	-2.208259	-3.283325
H	5.754720	-0.899940	-2.302481
N	-8.364361	1.028210	4.208590
C	-9.308499	0.670146	3.167478
C	-9.075564	1.281485	1.776265
O	-9.976826	1.178452	0.949208
C	-9.574265	-0.874186	3.088566
O	-9.724781	-1.392786	4.400092
C	-8.543692	-1.649603	2.280649
C	-7.300685	-1.999521	2.815107
C	-8.834408	-2.001857	0.961586
C	-6.338282	-2.602012	2.016244
C	-7.874066	-2.623063	0.170578
Cl	-8.258933	-3.039610	-1.493352
C	-6.604470	-2.886965	0.680482
O	-5.591157	-3.373251	-0.133096
N	-7.911711	1.948461	1.537585
C	-7.796805	2.763859	0.339937
C	-6.613365	2.503521	-0.606961
O	-6.615561	3.081728	-1.694392
C	-7.903797	4.288101	0.634535
C	-6.683016	4.887538	1.326207
O	-6.633467	5.077158	2.530399
N	-5.655097	5.213698	0.486821
H	-4.774232	5.469660	0.905604
H	-5.662110	4.919018	-0.478677
N	-5.628125	1.655176	-0.227692
C	-4.549617	1.359762	-1.157818
C	-3.319666	2.260911	-0.910300
O	-2.807011	2.364178	0.197940

C	-4.145943	-0.120187	-1.113923
C	-5.043194	-1.061343	-0.624988
C	-2.907045	-0.545631	-1.595802
C	-4.724460	-2.419850	-0.622024
C	-2.578222	-1.898064	-1.564427
C	-3.488109	-2.858758	-1.108578
O	-3.150297	-4.198783	-1.066159
N	-2.820288	2.876247	-2.010298
C	-1.452700	3.382561	-2.051059
C	-0.892287	2.999181	-3.444832
O	-1.665506	2.877124	-4.388659
C	-1.237730	4.887395	-1.889596
C	-0.282324	5.334929	-0.978495
C	0.194949	6.651854	-0.979145
C	-1.395763	7.143226	-2.755259
C	-1.804783	5.818361	-2.770147
C	-0.373562	7.556982	-1.892358
O	0.041797	8.849236	-1.972899
N	0.448056	2.864408	-3.584856
C	1.481122	3.001303	-2.574959
C	2.164028	4.381606	-2.749915
O	2.154845	4.936567	-3.839227
C	2.456403	1.791582	-2.603770
O	3.206579	1.863055	-3.808321
C	1.636476	0.518491	-2.453833
C	1.106741	-0.153352	-3.557136
C	1.242260	0.126662	-1.173575
C	0.136624	-1.135372	-3.384327
C	0.280539	-0.861948	-0.998919
Cl	-0.213586	-1.297771	0.620679
C	-0.317326	-1.465810	-2.111085
O	-1.343513	-2.380220	-1.982709
N	2.647666	4.914822	-1.613630
C	3.187760	6.269456	-1.563921
C	4.758734	6.318862	-1.797833
C	2.671587	6.962632	-0.296044
C	3.580353	7.433694	0.651274
C	1.280861	7.103602	-0.043328
C	3.141855	8.023117	1.834647
O	4.032167	8.487354	2.771678
C	0.872849	7.698981	1.168574
O	-0.450320	7.849726	1.476471
C	1.788070	8.153961	2.113869
O	5.273735	7.439134	-1.695579
O	5.284439	5.215574	-2.099666
C	-3.224911	-4.865091	-2.297742

C	-2.766870	-6.314708	-2.126587
O	-1.364020	-6.394773	-1.871258
C	-3.023674	-7.091641	-3.430031
O	-2.706104	-8.468634	-3.274499
C	-4.456672	-6.956583	-3.930584
O	-4.642454	-7.562933	-5.206136
C	-4.835176	-5.466174	-3.973714
O	-4.581800	-4.857491	-2.709345
C	-6.313997	-5.236252	-4.268736
O	-7.149007	-5.757122	-3.251749
H	-2.607389	-4.348955	-3.048301
H	-6.479501	-4.160497	-4.410595
H	-6.569400	-5.757804	-5.192643
C	-0.956698	-7.202899	-0.766365
C	0.485196	-7.657481	-1.006770
C	1.553269	-6.569395	-0.760233
N	2.890152	-7.208360	-0.801771
C	1.555981	-5.535319	-1.900112
C	1.227056	-5.914250	0.612228
C	-0.233962	-5.436357	0.697001
O	-1.110765	-6.560558	0.466653
C	-0.604960	-4.892247	2.064840
H	-1.620349	-8.071087	-0.706359
H	0.595390	-8.042623	-2.023861
H	0.659817	-8.492121	-0.322371
H	2.312284	-4.768601	-1.721543
H	0.588981	-5.052519	-2.015162
H	1.802044	-6.043247	-2.835918
H	-1.656906	-4.602668	2.069046
H	-0.000741	-4.016547	2.301902
H	-0.450951	-5.653316	2.835537
O	2.086052	-4.816585	0.928495
H	3.578161	-6.563835	-0.468258
H	2.888230	-8.023341	-0.222287
C	4.333910	0.993278	-3.894025
C	5.148140	1.356240	-5.130882
C	6.014659	2.648585	-5.035890
N	7.069680	2.627379	-6.070709
C	5.182978	3.909004	-5.287692
C	6.732443	2.686663	-3.646394
C	5.812337	2.280120	-2.477941
O	5.132111	1.010919	-2.735370
C	6.576588	2.097410	-1.179406
H	3.974190	-0.042160	-3.955660
H	4.484624	1.419071	-5.999862
H	5.822337	0.508156	-5.302163

H	5.838329	4.778107	-5.253831
H	4.381962	4.044913	-4.566603
H	4.726297	3.853789	-6.283414
H	5.893984	1.844046	-0.363914
H	7.084400	3.027985	-0.930509
H	7.323098	1.301363	-1.274029
O	7.339373	3.922719	-3.361277
H	-7.449771	1.115119	3.813659
H	-8.634637	1.899409	4.618424
H	-10.255776	1.107982	3.491155
H	-9.140712	-0.874018	4.969470
H	-10.538575	-0.986809	2.590194
H	-7.049611	-1.744533	3.835316
H	-5.349347	-2.816766	2.402625
H	-9.795696	-1.753158	0.530206
H	-5.996983	-0.735619	-0.241063
H	-2.185959	0.164843	-1.973521
H	-4.949116	1.589079	-2.149055
H	-5.502643	1.417305	0.762730
H	-8.671607	2.504607	-0.260070
H	-7.145482	1.927546	2.213007
H	-8.073174	4.791359	-0.319436
H	-8.763709	4.451055	1.283052
H	-6.951658	-5.264563	-2.444903
H	-4.235961	-4.971956	-4.759576
H	-5.142089	-7.469945	-3.253412
H	-3.894699	-7.319444	-5.762252
H	-1.757383	-8.517067	-3.118884
H	-2.360607	-6.646057	-4.193599
H	-3.337812	-6.747964	-1.300014
H	-0.422412	-4.673306	-0.062636
H	1.343356	-6.688060	1.384883
H	2.906827	-4.847770	0.407164
H	-3.229997	2.709617	-2.922517
H	1.636946	0.638177	-0.303550
H	1.408525	0.139457	-4.554542
H	-0.327464	-1.620358	-4.234738
H	3.125520	1.890450	-1.745451
H	5.064065	3.063135	-2.344313
H	7.544858	1.944037	-3.698640
H	6.670851	4.529593	-2.959808
H	7.614860	1.771596	-5.999745
H	6.640007	2.628946	-6.992514
H	0.775525	2.915508	-4.541496
H	1.023510	2.967560	-1.589901
H	2.602656	4.381861	-0.753254

H	2.772569	6.781879	-2.437411
H	1.442813	8.615386	3.029807
H	4.637227	7.394708	0.416329
H	4.922898	8.348414	2.430137
H	-0.971832	7.471749	0.754694
H	0.796869	8.956625	-1.375996
H	-1.815987	7.867821	-3.442241
H	-2.541677	5.495826	-3.497260
H	0.157580	4.637015	-0.276366
H	-0.915301	2.882371	-1.246347

Table S6. Cartesian coordinates for Vanco-PG complex

	x	*y*	*z*
C	2.034396	7.972911	3.761789
C	2.708747	6.764658	3.133183
O	3.921143	6.630815	3.102581
N	1.848650	5.843130	2.596607
C	2.323434	4.720870	1.778218
C	3.018080	3.644048	2.643051
C	3.625048	2.513937	1.807782
C	4.176192	1.358808	2.650037
C	4.836347	0.301385	1.759025
N	5.251067	-0.905519	2.471133
C	1.138186	4.122077	1.001799
O	1.189870	3.945258	-0.219509
N	0.079278	3.807287	1.771754
C	-1.115336	3.092637	1.334626
C	-2.314385	4.038330	1.159470
C	-1.418891	2.053853	2.436802
O	-0.909083	2.179543	3.553564
N	-2.283972	1.088214	2.100191
C	-2.808991	0.136439	3.073348
C	-1.871386	-1.064541	3.269883
C	-4.230398	-0.324809	2.675144
O	-4.828609	-1.010014	3.539319
O	-4.676998	0.012377	1.546131
N	7.237008	0.244622	-2.525973
C	6.500607	-0.609310	-1.596739
C	7.418637	-1.528717	-0.786194
O	6.976978	-2.482432	-0.133712
N	8.722746	-1.200163	-0.849610

C	9.800818	-2.032909	-0.352501
C	10.814464	-1.350292	0.574216
O	11.980759	-1.701564	0.553623
N	10.328517	-0.389316	1.410534
C	11.201593	0.273801	2.373343
C	11.225557	-0.413089	3.753071
O	12.252988	-0.822872	4.262412
N	9.996629	-0.530969	4.329739
C	9.746416	-1.342924	5.509087
C	8.383479	-2.034903	5.384007
O	7.610966	-2.109203	6.317315
N	8.100800	-2.522465	4.121536
C	6.737754	-2.438354	3.636211
C	6.483950	-1.069127	2.971789
O	7.366694	-0.203599	2.919966
H	-2.897625	0.657992	4.028864
H	-0.898243	-0.721123	3.626747
H	-2.301988	-1.746165	4.005081
H	-1.730303	-1.600439	2.326832
H	-2.677588	1.056575	1.162587
H	-0.907162	2.589673	0.392008
H	-2.072771	4.839606	0.460294
H	-3.170635	3.482662	0.775863
H	-2.590226	4.488255	2.116927
H	0.194950	3.772202	2.781707
H	3.031573	5.092440	1.033587
H	0.879770	6.107357	2.498944
H	1.070034	7.723188	4.210711
H	1.868104	8.733166	2.992751
H	2.698000	8.381191	4.523004
H	3.798582	4.145240	3.218318
H	2.294368	3.234806	3.356327
H	2.868489	2.111876	1.125069
H	4.422421	2.924887	1.176002
H	4.910057	1.726738	3.374428
H	3.363565	0.897155	3.220957
H	4.140314	-0.013587	0.977676
H	5.719787	0.733035	1.282869
H	4.675544	-1.734851	2.361502
H	6.522007	-3.228568	2.914797
H	6.067580	-2.565148	4.487612
H	8.787057	-2.261798	3.426599
H	10.561126	-2.066672	5.604247
H	9.712454	-0.754512	6.428880
H	9.177550	-0.154971	3.858023
H	12.218632	0.240508	1.989495

H	10.884852	1.314897	2.481625
H	9.340191	-0.173677	1.402016
H	9.351733	-2.873866	0.181472
H	10.388060	-2.440745	-1.178209
H	8.907352	-0.419454	-1.477617
H	5.964433	0.017261	-0.880968
H	5.745869	-1.253713	-2.062853
H	7.322092	-0.198534	-3.434282
H	6.765157	1.136866	-2.672271
N	-8.012895	0.456400	6.284260
C	-7.527766	0.464606	4.902254
C	-8.313001	-0.528345	4.024560
O	-9.534322	-0.590067	4.107663
C	-7.712199	1.864451	4.281919
C	-6.903061	2.992008	4.946733
C	-7.372271	4.351057	4.405559
C	-5.393310	2.821876	4.729191
H	-4.835502	3.642946	5.191204
H	-5.157514	2.812237	3.660132
H	-5.021374	1.889832	5.161237
H	-6.813291	5.173696	4.864462
H	-8.435963	4.512827	4.609839
H	-7.229451	4.402953	3.321709
C	-7.702940	-0.773155	7.008667
N	-7.572254	-1.292697	3.158347
C	-8.245706	-2.109704	2.167144
C	-8.139422	-1.655731	0.702193
O	-8.879693	-2.192342	-0.116929
C	-7.939597	-3.640265	2.326832
O	-7.995896	-3.990016	3.700224
C	-6.642617	-4.094217	1.672727
C	-5.400924	-3.892051	2.281399
C	-6.688036	-4.707932	0.419805
C	-4.229494	-4.210057	1.607587
C	-5.512487	-5.042838	-0.243348
Cl	-5.596550	-5.797956	-1.828601
C	-4.274653	-4.755189	0.328092
O	-3.095877	-4.949707	-0.377482
N	-7.273232	-0.652745	0.385790
C	-7.364068	-0.026486	-0.922638
C	-6.096733	0.030281	-1.791868
O	-6.220967	0.410880	-2.956576
C	-8.026856	1.380602	-0.873983
C	-7.154582	2.471578	-0.260022
O	-7.267460	2.832054	0.900066
N	-6.248391	3.027534	-1.118683

H	-5.550247	3.640819	-0.727155
H	-6.076807	2.618892	-2.025680
N	-4.906630	-0.341902	-1.263317
C	-3.725915	-0.353130	-2.112652
C	-2.918251	0.957356	-1.984518
O	-2.561631	1.392608	-0.895916
C	-2.828171	-1.564783	-1.827900
C	-3.367751	-2.689038	-1.215695
C	-1.486043	-1.575310	-2.210862
C	-2.589113	-3.825231	-0.992244
C	-0.702324	-2.698293	-1.960107
C	-1.244226	-3.849741	-1.377755
O	-0.457374	-4.956494	-1.117863
N	-2.587293	1.552086	-3.157025
C	-1.489350	2.508366	-3.248481
C	-0.724891	2.163822	-4.552527
O	-1.330192	1.639786	-5.481274
C	-1.835078	3.995628	-3.321506
C	-1.172949	4.881061	-2.472515
C	-1.195237	6.267556	-2.670545
C	-2.716468	5.896837	-4.534631
C	-2.626576	4.525580	-4.349270
C	-1.976440	6.768616	-3.726519
O	-2.041583	8.098819	-4.000832
N	0.582615	2.505556	-4.641622
C	1.419761	3.146585	-3.644564
C	1.579905	4.641685	-4.020380
O	1.457549	4.998067	-5.183431
C	2.759867	2.380884	-3.462226
O	3.525408	2.550641	-4.647109
C	2.435918	0.931482	-3.130847
C	2.264671	-0.033287	-4.125413
C	2.109942	0.605946	-1.813363
C	1.695782	-1.265638	-3.820408
C	1.551073	-0.629180	-1.505322
Cl	1.121730	-0.984172	0.152040
C	1.292901	-1.557207	-2.520762
O	0.651168	-2.753745	-2.271438
N	1.754851	5.466592	-2.972385
C	1.773890	6.917609	-3.125323
C	3.238145	7.499540	-3.331304
C	0.950373	7.546155	-1.993427
C	1.557933	8.441427	-1.113355
C	-0.414871	7.207584	-1.795096
C	0.850041	8.990709	-0.046938
O	1.443610	9.871573	0.823668

C	-1.098642	7.777373	-0.700814
O	-2.408606	7.479968	-0.448133
C	-0.479208	8.659879	0.179927
O	3.312920	8.732795	-3.395990
O	4.142366	6.631063	-3.444650
C	-0.196691	-5.769196	-2.230468
C	0.731258	-6.915472	-1.823713
O	2.047469	-6.445596	-1.529783
C	0.866878	-7.906128	-2.993648
O	1.639357	-9.038525	-2.616679
C	-0.478240	-8.370404	-3.538977
O	-0.339066	-9.174236	-4.706750
C	-1.356297	-7.139889	-3.824096
O	-1.432457	-6.310983	-2.666323
C	-2.793431	-7.504696	-4.182392
O	-3.464333	-8.145491	-3.113709
H	0.252910	-5.174418	-3.039892
H	-3.318402	-6.592951	-4.495265
H	-2.776017	-8.206600	-5.017784
C	2.629446	-6.888932	-0.303193
C	4.152669	-6.819149	-0.438928
C	4.743253	-5.395142	-0.344567
N	6.218857	-5.505620	-0.257118
C	4.465901	-4.599929	-1.632727
C	4.102271	-4.718275	0.900532
C	2.564824	-4.795183	0.878542
O	2.164054	-6.180944	0.809836
C	1.921842	-4.237514	2.135625
H	2.314369	-7.921090	-0.121093
H	4.469493	-7.275901	-1.380178
H	4.559257	-7.429786	0.371829
H	4.885123	-3.594494	-1.561373
H	3.402308	-4.521227	-1.842481
H	4.946692	-5.109309	-2.471560
H	0.838532	-4.351043	2.072263
H	2.156227	-3.178571	2.244402
H	2.276550	-4.775788	3.019573
O	4.489311	-3.351450	1.057872
H	6.605966	-4.615950	-0.014973
H	6.461955	-6.176697	0.443281
H	-7.103826	2.951865	6.023247
H	-7.466925	1.829952	3.215495
H	-8.781447	2.103378	4.338476
H	-6.471253	0.186704	4.903158
H	-8.098505	-0.703644	8.026236
H	-8.112529	-1.690393	6.551271

H	-6.617919	-0.889720	7.080905
H	-9.023660	0.562661	6.238404
H	-6.539309	-1.199822	3.187809
H	-9.308416	-2.004175	2.398039
H	-7.679021	-3.221033	4.192875
H	-8.760274	-4.162755	1.832419
H	-5.335166	-3.423973	3.253748
H	-3.261215	-3.995972	2.043485
H	-7.639160	-4.886988	-0.065289
H	-4.401357	-2.680800	-0.907801
H	-1.037285	-0.711969	-2.680616
H	-4.103971	-0.424578	-3.135738
H	-4.780826	-0.377698	-0.245399
H	-8.041581	-0.665856	-1.492446
H	-6.603273	-0.300402	1.071902
H	-8.290472	1.650167	-1.898610
H	-8.935418	1.309689	-0.277274
H	-3.516639	-7.507643	-2.390538
H	-0.913291	-6.576860	-4.665305
H	-0.986499	-8.997440	-2.803861
H	0.313777	-8.756479	-5.278558
H	2.528761	-8.717867	-2.435642
H	1.384936	-7.361689	-3.803858
H	0.289873	-7.406404	-0.951318
H	2.176476	-4.266016	0.004484
H	4.426208	-5.281676	1.787633
H	5.304970	-3.155549	0.565173
H	-2.840450	1.123032	-4.039972
H	2.229989	1.341539	-1.026686
H	2.518072	0.206918	-5.149972
H	1.500155	-1.999185	-4.593343
H	3.282902	2.833932	-2.616421
H	2.976149	2.943541	-5.329417
H	0.942574	2.537977	-5.587341
H	0.930203	3.086985	-2.676232
H	1.836355	5.078962	-2.039958
H	1.271822	7.117805	-4.077055
H	-1.034427	9.087706	1.004399
H	2.574544	8.755548	-1.317155
H	2.348973	10.018548	0.526899
H	-2.705505	6.842168	-1.112111
H	-1.421543	8.554431	-3.412675
H	-3.312699	6.316739	-5.335732
H	-3.143441	3.860126	-5.031733
H	-0.568997	4.494987	-1.660251
H	-0.872496	2.354125	-2.364033

Table S7. Cartesian coordinates for Des-Vanco-PG complex

	x	*y*	*z*
C	3.122686	7.575430	4.131396
C	3.536587	6.333278	3.359702
O	4.703884	6.057263	3.136123
N	2.495984	5.553686	2.928104
C	2.696790	4.431556	2.003361
C	3.370216	3.231432	2.707786
C	3.697123	2.086669	1.745558
C	4.216511	0.827404	2.446961
C	4.596490	-0.248478	1.424245
N	4.956094	-1.535252	2.016365
C	1.342555	4.024541	1.397905
O	1.188465	3.913275	0.177493
N	0.379120	3.795042	2.310227
C	-0.948377	3.254821	2.036691
C	-2.030136	4.345723	2.086325
C	-1.213524	2.198238	3.131834
O	-0.530940	2.197629	4.159909
N	-2.234771	1.364033	2.896703
C	-2.725287	0.427610	3.902218
C	-1.929148	-0.886255	3.904453
C	-4.237166	0.163457	3.712062
O	-4.781847	-0.493733	4.631619
O	-4.800779	0.615983	2.679892
N	6.303749	-0.345874	-3.180467
C	5.611829	-1.157495	-2.181728
C	6.516205	-2.225217	-1.559516
O	6.059318	-3.155115	-0.883562
N	7.827294	-2.052020	-1.811932
C	8.852833	-3.034993	-1.520632
C	10.071882	-2.532993	-0.736946
O	11.167838	-3.019569	-0.951853
N	9.842312	-1.570413	0.201022
C	10.926428	-1.072989	1.041408
C	11.068591	-1.835942	2.373224
O	12.100043	-2.394338	2.700735
N	9.934605	-1.838818	3.128720
C	9.762108	-2.681399	4.300489
C	8.319013	-3.196931	4.361986
O	7.691360	-3.231735	5.400350

N	7.791418	-3.573804	3.140893
C	6.393063	-3.299405	2.876661
C	6.218997	-1.873745	2.312878
O	7.186863	-1.118561	2.157982
H	-2.603299	0.900252	4.879261
H	-0.877979	-0.682860	4.118859
H	-2.328270	-1.552547	4.670809
H	-1.999667	-1.380110	2.931020
H	-2.764724	1.433660	2.031060
H	-0.948856	2.785554	1.054537
H	-1.795754	5.151159	1.389470
H	-2.997642	3.919503	1.819207
H	-2.100535	4.769668	3.091590
H	0.638842	3.688392	3.287870
H	3.327257	4.757862	1.172677
H	1.564901	5.937153	2.992281
H	2.212311	7.417551	4.714644
H	2.941435	8.393046	3.427327
H	3.938919	7.856930	4.795497
H	4.285130	3.601713	3.173761
H	2.715011	2.871453	3.508488
H	2.802246	1.817949	1.173617
H	4.436941	2.434841	1.013934
H	5.091019	1.062617	3.062252
H	3.446206	0.434300	3.118926
H	3.757307	-0.432457	0.748894
H	5.446564	0.100968	0.833677
H	4.269987	-2.282057	1.965584
H	5.973404	-4.015349	2.167624
H	5.846866	-3.393781	3.816266
H	8.393752	-3.357655	2.358393
H	10.483247	-3.501722	4.240095
H	9.940870	-2.146980	5.236353
H	9.108785	-1.341008	2.804703
H	11.862207	-1.205664	0.503328
H	10.764289	-0.009420	1.237527
H	8.899292	-1.237899	0.354345
H	8.385305	-3.845435	-0.956171
H	9.253606	-3.462065	-2.442570
H	8.013785	-1.264235	-2.430475
H	5.272519	-0.513423	-1.367824
H	4.720274	-1.679075	-2.549465
H	6.195289	-0.743102	-4.107343
H	5.932595	0.603354	-3.217298
N	-7.565048	-0.423629	4.670617
C	-8.477206	-1.095844	3.765291

C	-8.533966	-0.574439	2.320167
O	-9.450330	-0.970629	1.605868
C	-8.347692	-2.658670	3.816510
O	-8.242339	-3.077498	5.167504
C	-7.231197	-3.226258	2.952007
C	-5.897051	-3.209477	3.367852
C	-7.540740	-3.757114	1.698652
C	-4.889337	-3.626163	2.508617
C	-6.529544	-4.191656	0.848649
Cl	-6.944557	-4.838943	-0.732199
C	-5.193877	-4.087547	1.231631
O	-4.168009	-4.380893	0.344654
N	-7.604359	0.334171	1.911955
C	-7.809159	1.040938	0.658570
C	-6.688946	0.995455	-0.394036
O	-6.936259	1.454475	-1.509818
C	-8.273023	2.512103	0.863634
C	-7.187396	3.453739	1.376250
O	-7.079114	3.757879	2.552798
N	-6.356311	3.945645	0.409352
H	-5.535339	4.447442	0.711051
H	-6.375368	3.572197	-0.528330
N	-5.490333	0.453750	-0.071369
C	-4.460731	0.350100	-1.093504
C	-3.483085	1.544950	-1.041984
O	-2.915583	1.871116	-0.006015
C	-3.691868	-0.974641	-0.998411
C	-4.272458	-2.059535	-0.353577
C	-2.434282	-1.123714	-1.585209
C	-3.619952	-3.291705	-0.297601
C	-1.771039	-2.344968	-1.502562
C	-2.361818	-3.454821	-0.887882
O	-1.692076	-4.660889	-0.796076
N	-3.257992	2.162171	-2.227894
C	-2.073487	2.983824	-2.451831
C	-1.562293	2.625758	-3.871026
O	-2.361072	2.232175	-4.714007
C	-2.234418	4.503497	-2.413017
C	-1.345895	5.253058	-1.643682
C	-1.221126	6.641324	-1.782354
C	-3.038197	6.563074	-3.400539
C	-3.096629	5.182546	-3.284281
C	-2.081123	7.292127	-2.684175
O	-2.016917	8.634112	-2.893661
N	-0.250030	2.813087	-4.148811
C	0.801159	3.290952	-3.269889

C	1.092084	4.775241	-3.608202
O	0.843782	5.209939	-4.723572
C	2.045182	2.361152	-3.327408
O	2.640577	2.505857	-4.609353
C	1.592803	0.944316	-3.005771
C	1.153828	0.065730	-3.998069
C	1.428443	0.584979	-1.667167
C	0.484906	-1.105144	-3.656088
C	0.769448	-0.590020	-1.323852
Cl	0.550715	-0.985707	0.365168
C	0.246786	-1.420584	-2.321846
O	-0.497326	-2.544044	-2.022144
N	1.524889	5.511690	-2.569120
C	1.705114	6.956369	-2.667214
C	3.184160	7.369724	-3.075474
C	1.146400	7.612884	-1.397857
C	1.987302	8.376942	-0.588990
C	-0.205774	7.429325	-1.003156
C	1.522124	8.944818	0.594732
O	2.346063	9.696815	1.395872
C	-0.640533	8.013037	0.205116
O	-1.925155	7.860539	0.646468
C	0.210490	8.762982	1.012308
O	3.404541	8.586827	-3.103483
O	3.943559	6.407219	-3.361154
C	-1.705798	-5.433670	-1.966098
C	-0.880798	-6.704238	-1.752746
O	0.513458	-6.412952	-1.648859
C	-1.048327	-7.635135	-2.966791
O	-0.378462	-8.871626	-2.758171
C	-2.507742	-7.902966	-3.314137
O	-2.647724	-8.649243	-4.519411
C	-3.254959	-6.561866	-3.411924
O	-3.051493	-5.797706	-2.225339
C	-4.764047	-6.730842	-3.556533
O	-5.343840	-7.347066	-2.422108
H	-1.310162	-4.851464	-2.812015
H	-5.209677	-5.746357	-3.748797
H	-4.960860	-7.380617	-4.410880
C	1.210898	-6.992498	-0.545467
C	2.693173	-7.097853	-0.913246
C	3.467335	-5.762563	-0.856996
N	4.913324	-6.053707	-1.004006
C	3.104134	-4.867178	-2.054916
C	3.110653	-5.086508	0.497628
C	1.589981	-4.977450	0.711647

O	1.010639	-6.299026	0.652724
C	1.217980	-4.419987	2.073824
H	0.797957	-7.988269	-0.356511
H	2.805312	-7.534401	-1.909139
H	3.135297	-7.798333	-0.199543
H	3.653635	-3.924922	-2.010878
H	2.039868	-4.649816	-2.093822
H	3.385658	-5.381398	-2.977191
H	0.131804	-4.399206	2.175091
H	1.598616	-3.404724	2.185558
H	1.629350	-5.046741	2.870614
O	3.687354	-3.787150	0.645447
H	5.442118	-5.232161	-0.790844
H	5.170977	-6.788183	-0.376185
H	-6.742128	-0.150286	4.172534
H	-8.009736	0.387585	5.050333
H	-9.471422	-0.877318	4.162256
H	-7.760360	-2.381556	5.634206
H	-9.292639	-3.049897	3.435766
H	-5.627949	-2.809282	4.335484
H	-3.847575	-3.554915	2.796819
H	-8.568572	-3.792870	1.360573
H	-5.239004	-1.945346	0.111134
H	-1.954643	-0.294738	-2.085365
H	-4.993244	0.383319	-2.047476
H	-5.219577	0.344726	0.912535
H	-8.639826	0.521061	0.176678
H	-6.800212	0.563783	2.498782
H	-8.650213	2.869708	-0.096504
H	-9.083899	2.516281	1.590985
H	-5.206133	-6.750156	-1.675584
H	-2.874568	-6.008512	-4.289415
H	-2.976090	-8.505855	-2.533705
H	-2.039814	-8.280470	-5.169136
H	0.561454	-8.670665	-2.705204
H	-0.592075	-7.110971	-3.826049
H	-1.245782	-7.188119	-0.841948
H	1.145941	-4.356264	-0.070508
H	3.489128	-5.734665	1.301341
H	4.438537	-3.662302	0.040000
H	-3.692553	1.817949	-3.076681
H	1.757060	1.254491	-0.880951
H	1.279903	0.332347	-5.039534
H	0.084488	-1.764370	-4.416926
H	2.741889	2.698987	-2.556260
H	2.050055	3.000225	-5.182488

H	-0.034100	2.856474	-5.136990
H	0.458066	3.234838	-2.240314
H	1.694736	5.064161	-1.676264
H	1.096163	7.269571	-3.521046
H	-0.156525	9.206105	1.928824
H	2.993684	8.578312	-0.935905
H	3.208239	9.751174	0.968024
H	-2.396451	7.302073	0.012590
H	-1.263219	8.977682	-2.391806
H	-3.689079	7.096651	-4.082607
H	-3.789895	4.624061	-3.903321
H	-0.681476	4.751443	-0.950491
H	-1.356181	2.706297	-1.680468
